# Predictive Factors of Successful Decannulation in Tracheostomy Patients: A Scoping Review

**DOI:** 10.3390/jcm14113798

**Published:** 2025-05-28

**Authors:** Andrea Calderone, Serena Filoni, Rosaria De Luca, Francesco Corallo, Rosalia Calapai, Alessio Mirabile, Fabrizia Caminiti, Valeria Conti-Nibali, Angelo Quartarone, Rocco Salvatore Calabrò, Carmela Rifici

**Affiliations:** 1 IRCCS Centro Neurolesi Bonino-Pulejo, S.S. 113 Via Palermo, C.da Casazza, 98124 Messina, Italy; andrea.calderone95@gmail.com (A.C.); rosaria.deluca@irccsme.it (R.D.L.); francesco.corallo@irccsme.it (F.C.); rosalia.calapai@irccsme.it (R.C.); alessio.mirabile@irccsme.it (A.M.); fabrizia.caminiti@irccsme.it (F.C.); valeria.continibali@irccsme.it (V.C.-N.); angelo.quartarone@irccsme.it (A.Q.); roccos.calabro@irccsme.it (R.S.C.); carmela.rifici@irccsme.it (C.R.); 2 Unit of Neuro-Rehabilitation, IRCCS “Casa Sollievo della Sofferenza”, 71013 San Giovanni Rotondo, Italy

**Keywords:** tracheostomy, decannulation, treatment outcome, scoping review, airway management

## Abstract

**Background/Objective**: Tracheostomy (TCT) creates an artificial airway, essential for overcoming obstructions or enabling long-term ventilation. Decannulation represents a critical step in recovery, with its success strongly influenced by the underlying indication for tracheostomy and the patient’s clinical profile. Successful decannulation requires careful assessment of multiple factors, including respiratory function and underlying pathology. This scoping review aims to identify and categorize these predictive factors, crucial for optimizing decannulation protocols and patient outcomes. **Methods**: A scoping review was conducted using the PubMed, Web of Science, Cochrane Library, Embase, EBSCOhost, and Scopus databases (22 February 2025–3 March 2025) to identify studies regarding predictors of successful decannulation. Studies examining physiological, clinical, and demographic factors associated with decannulation outcomes were included. Data were extracted using a standardized form and synthesized narratively. **Results**: Fifty studies reported a male representation averaging 67% of the total patient population, comprising 2238 males and 1281 females aged 50–70 with acquired brain injuries, employing retrospective and prospective designs. Positive decannulation outcomes correlate with strong cough, effective secretion management, younger age, and robust neurological status. Adverse events were generally mild, with recannulation being infrequent. Conversely, advanced age, chronic lung disease, a high body mass index, and prolonged mechanical ventilation negatively influence decannulation success. **Conclusions**: It was highlighted that successful decannulation is the result of various physiological, clinical, and demographic factors. Significantly, strong respiratory function, demonstrated by powerful cough reflexes and efficient secretion control, stands out as a fundamental predictive factor.

## 1. Introduction

A tracheostomy (TCT) is a medical procedure in which an opening, or stoma, is created in front of the trachea through an incision in the neck to provide direct access to the lungs [1]. The stoma provides access to place a TCT tube, permitting respiration [2]. The primary indication of a TCT is to circumvent an upper airway obstruction or compromise [3]. It is also performed frequently to permit long-term mechanical ventilation in a patient with respiratory failure [4]. The procedure permits effective clearance of secretions through the lungs, which is particularly important in patients that cannot effectively cough [5]. In some cases, a TCT can be used to permit the delivery of oxygen to the lungs more effectively or more securely [6]. TCT remains a relatively frequent intervention in adult populations, with incidence estimates in the United States ranging from 28.4 to 39.7 per 100,000 individuals annually [7,8]. In contrast, pediatric cases are notably rarer, likely reflecting distinct etiologies and clinical trajectories, averaging between 6.0 and 7.1 per 100,000 children under 18 years of age [9,10,11]. TCT can be performed in two main ways: surgical TCT and percutaneous TCT. The first one is an open surgery that is traditionally performed in a theater of operations with a direct incision through the skin and tissues of the neck to expose the trachea [12]. Percutaneous TCT is a minimally invasive procedure that can often be performed at the bedside, particularly in intensive care units. This is achieved with a small cut, using a needle and dilators of gradually increasing sizes to enlarge the tracheal opening [13,14]. The increasing use of percutaneous TCT, favored for its efficiency and lower cost, is reshaping clinical trends [15]. TCT may be temporary or permanent, depending on whether the underlying condition is reversible or chronic, such as in severe neurological impairment [16,17]. As previously described, a TCT serves to bypass upper airway obstructions or facilitate long-term ventilation. Whether it is a surgical or percutaneous procedure, the goal is to establish a reliable airway. However, the need for this artificial airway is not always permanent [18]. The process of removing the TCT tube, known as decannulation, is a critical step in a patient’s recovery. Decannulation is essentially the reverse of the TCT procedure, but it is not simply a matter of pulling out the tube [19]. It is a carefully managed process that requires thorough assessment and preparation [20]. Given the range of TCT indications, from reversible obstructions to chronic respiratory failure, decannulation pathways vary accordingly [21]. In transient cases, removal may follow a straightforward course once airway patency is restored [22], while patients with chronic neurological impairment require a comprehensive assessment of autonomous respiratory capacity before decannulation can be safely attempted [23,24]. The shift towards percutaneous TCTs, as mentioned, might also influence decannulation practices. The minimally invasive nature of these procedures could potentially facilitate a smoother transition to natural breathing compared to traditional surgical TCTs [25]. Drawing from this evolving clinical experience, we outlined in Table 1 the typical decannulation pathway commonly adopted in specialized settings. These structured steps offer a practical lens through which to interpret clinical decision-making and frame the identification of key predictive factors.

Despite its clinical relevance, the decannulation process remains poorly understood, with significant gaps in identifying reliable predictors of success. The heterogeneity of TCT indications complicates the development of broadly applicable models. While factors such as cough strength, secretion management, and ventilatory parameters have been explored, a comprehensive and integrative framework is still lacking [37,38]. This scoping review addresses the following research question: What are the predictive factors associated with successful decannulation in patients with TCT? To answer this question, we aim to comprehensively map the existing literature to identify and categorize the predictive factors associated with successful decannulation in patients with TCT. This review will explore a range of potential predictors, including but not limited to physiological, clinical, and demographic factors, to provide a broad understanding of the current state of evidence and to highlight areas for future research. The theoretical basis for this scoping review is multidisciplinary in nature, combining physiological, clinical, and psychosocial models. Physiological models have been directed toward assessing respiratory function, i.e., parameters such as vital capacity, cough capacity, secretion clearance, and parameters of ventilation [39]. Clinical models have aimed at evaluating patient demographics, underlying conditions, TCT techniques, and TCT durations [40]. This intersection of different models will provide a more cohesive understanding of predictive factors and help in developing an overarching conceptual framework for successful decannulation.

## 2. Materials and Methods

### 2.1. PICO Evaluation

To structure our search strategy, we adapted the PICO framework (Population, Intervention, Comparison, Outcome). The population encompassed both adult and pediatric individuals requiring TCT. The intervention in this context was the analysis of various physiological, clinical, and demographic factors potentially predictive of successful decannulation. As this was a scoping review, rather than an interventional study, a direct comparison group was not applicable. The primary outcome was the identification and categorization of key predictors associated with successful decannulation.

### 2.2. Search Strategy

A structured methodology was employed in this scoping review to evaluate research studies within the defined period of 2014 to 2025. The investigation included all relevant research available within this time frame, ensuring a thorough understanding of the current state of evidence regarding predictors of successful decannulation and preventing the omission of significant data. We performed an extensive literature search (between 22 February 2025 and 3 March 2025) through the PubMed, Web of Science, Cochrane Library, Embase, EBSCOhost, and Scopus databases, employing the following keywords: (“tracheostomy” [MeSH Terms] OR “tracheostomy” [All Fields] OR “tracheostomies” [All Fields]) AND (“decannulate” [All Fields] OR “decannulated” [All Fields] OR “decannulating” [All Fields] OR “decannulation” [All Fields] OR “decannulations” [All Fields]) AND (“predictor” [All Fields] OR “predictors” [All Fields]). These databases were meticulously chosen to encompass the widest possible spectrum of the peer-reviewed literature in areas pertinent to this review. PubMed was selected for its comprehensive indexing of biomedical research, particularly its extensive coverage of airway management and respiratory rehabilitation studies. Web of Science was included for its multidisciplinary scope and citation tracking features, enabling the identification of key studies related to decannulation. The Cochrane Library was chosen to allow us access to high-quality evidence, including systematic reviews and randomized controlled trials relevant to decannulation outcomes. Embase was utilized for its broad coverage of clinical and pharmacological research, particularly in respiratory and critical care medicine. EBSCOhost was chosen for its range of health sciences databases. Scopus was incorporated for its extensive multidisciplinary coverage and advanced citation analysis tools, reflecting the diverse range of studies related to decannulation predictors. By employing these databases, we aimed to maximize the comprehensiveness and validity of our search strategy, minimize the risk of missing pertinent studies, and ensure the inclusion of high-quality, diverse evidence on decannulation predictors.

### 2.3. Inclusion Criteria

This scoping review considered studies investigating predictors of successful decannulation in both adult and pediatric populations, identified through a comprehensive search across six major databases: PubMed, Web of Science, Cochrane Library, Embase, EBSCOhost, and Scopus. To ensure a comprehensive understanding of these predictors, eligible studies were required to include participants undergoing TCT. Studies were considered if they examined physiological, clinical, and demographic factors associated with decannulation outcomes. Only studies that specifically addressed predictors of successful decannulation were included, focusing on outcomes such as time to decannulation, decannulation success rates, and associated complications. Furthermore, studies had to clearly describe the assessed predictors, including methodology, participant demographics, and relevant clinical parameters, to ensure clarity and reproducibility. To maintain consistency and facilitate robust evidence evaluation, only full-text articles published in English were included.

### 2.4. Exclusion Criteria

Studies were excluded if they did not specifically focus on predictors of successful decannulation in adult and pediatric populations undergoing TCT. Studies investigating non-decannulation-related factors, such as general airway management or alternative ventilation techniques, were omitted as they did not align with the objectives of this review. Additionally, studies that did not address relevant outcomes related to decannulation, such as time to decannulation, success rates, or associated complications, were excluded. Studies that failed to clearly define participant groups or included a mix of individuals with different airway management protocols without specifically isolating those with TCT were also excluded to maintain consistency in the analysis. Furthermore, study protocols, conference abstracts, editorials, and reviews, whether systematic, narrative, or integrative, were not included. Finally, studies published in languages other than English or those without full-text access were excluded to ensure a thorough and methodologically rigorous evaluation.

### 2.5. Data Extraction

**Figure 1:**
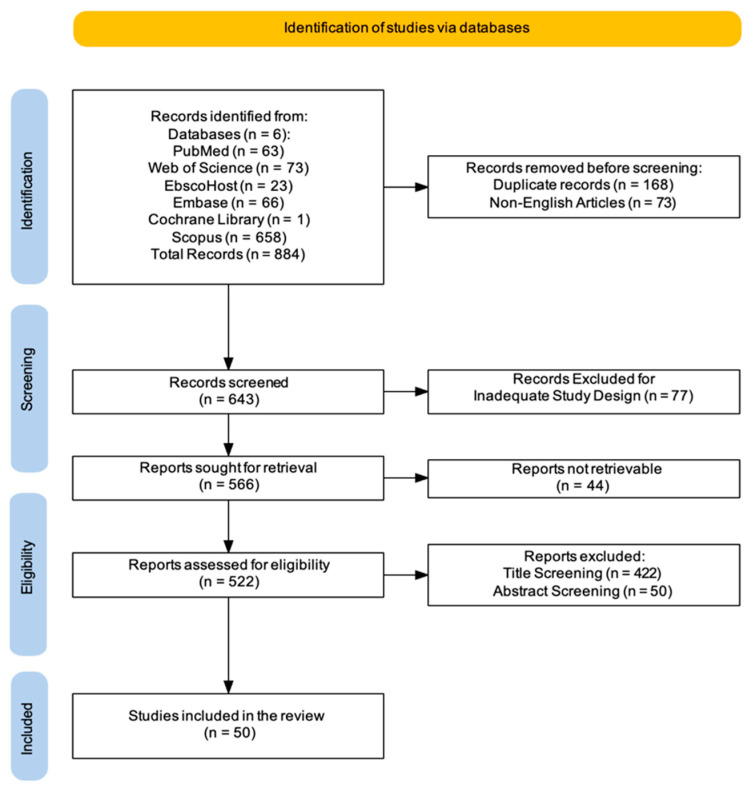
PRISMA 2020 flow diagram of evaluated studies.

Two reviewers (A.C. and S.F.) performed independent searches to improve transparency and accuracy in locating pertinent studies. The search strategy was iteratively refined by testing different combinations of keywords, Boolean operators, and controlled vocabulary (e.g., MeSH terms) to maximize sensitivity and specificity. The PRISMA flowchart was employed to depict the process (identification, screening, eligibility, and inclusion) of choosing relevant studies, as shown in Figure 1 [41]. Additionally, two researchers (A.C. and S.F.) screened all articles based on titles, abstracts, and full texts, conducting independent data extraction, article gathering, and cross-validation to minimize bias risks (e.g., missing results bias, publication bias, time-lag bias, or language bias). The researchers (A.C. and S.F.) reviewed complete-text articles considered suitable for the study, and if there were disagreements regarding the inclusion and exclusion criteria, a final decision was reached by a third researcher (R.S.C.). Discrepancies between reviewers during the screening or data extraction process were also resolved through discussion, with unresolved cases adjudicated by a third reviewer (R.S.C.). Moreover, the concordance between the two evaluators (A.C. and S.F.) was evaluated through the kappa statistic. The kappa score, which has a recognized threshold for significant agreement established at >0.61, was understood to indicate substantial alignment among the reviewers [42]. This standard guarantees a strong assessment of inter-rater reliability, highlighting the attainment of a significant degree of consensus in the data extraction procedure. The process of data extraction and organization was simplified through the use of Microsoft Excel, reducing human error and enhancing efficiency. The software facilitated effective handling of extensive datasets, permitting reviewers to methodically document study attributes, evaluations of bias risk, and outcome information. Custom extraction sheets were created in the software to guarantee uniformity and compliance with the established inclusion/exclusion criteria. Moreover, the software offered functionalities like tagging, filtering, and sorting, which helped in resolving discrepancies and sped up the cross-validation procedure. The collection of articles was later improved for relevance, evaluated, and summarized, with key topics emphasized from the summary based on the inclusion/exclusion criteria. This scoping review has been registered on Open OSF with the following DOI number: DOI 10.17605/OSF.IO/4A36M.

#### Data Synthesis

Given the heterogeneity of study designs and the diverse range of factors potentially influencing decannulation outcomes, data synthesis was conducted using narrative methods to systematically analyze the identified studies and extract relevant predictors of successful decannulation. This method enabled the identification of common themes and variations across research, providing a comprehensive overview of the factors associated with decannulation outcomes. A multidisciplinary team, through regular discussions and consensus meetings, ensured impartial interpretation of the data. Frequent discussions and consensus meetings among reviewers aided in reducing potential biases in qualitative evaluations and ensured uniformity in categorizing and interpreting results. Table 2 outlines the methodology used in this scoping review.

## 3. Results

A comprehensive literature search was carried out using six electronic databases. This initial search yielded a total of 884 records. Before formal screening commenced, 168 duplicate records were identified and removed, along with 73 non-English articles. This left 643 records for title and abstract screening. Following this initial screening phase, 77 records were excluded based on inadequate study design, leaving 566 reports for full-text retrieval. However, despite efforts to obtain these articles (e.g., emailing corresponding authors, consulting libraries, exploring open-access sources, checking institutional resources, and using research networks), 44 reports could not be retrieved. The remaining 522 reports underwent a thorough assessment of eligibility. During this stage, 422 reports were further excluded based on title screening, and an additional 50 were excluded after abstract screening. This rigorous selection process resulted in a final set of 50 studies [43,44,45,46,47,48,49,50,51,52,53,54,55,56,57,58,59,60,61,62,63,64,65,66,67,68,69,70,71,72,73,74,75,76,77,78,79,80,81,82,83,84,85,86,87,88,89,90,91,92] that met the pre-defined inclusion criteria and were therefore included in this review (Figure 1).

### 3.1. Demographic and Aetiological Characteristics: Age, Sex, Diagnosis, Observation Periods, and Their Geographic Distribution

The demographic profiles of patients undergoing TCT decannulation across various international studies consistently reveal a significant male predominance. Notably, studies conducted in Spain [43], Italy [50], China [46,47,58,80,83], Germany [48,73], and the United States [68,74] reported male representation ranging from approximately 60% to 70% of the patient population, totaling 2238 males and 1281 females across the included studies where sex was explicitly reported. The mean age of patients in these studies fell within a range of 50 to 70 years. However, pediatric populations, as observed in studies from the United States [69,87,89] and Japan [85], demonstrated significantly lower median ages, typically under 10 years. The primary diagnoses necessitating TCT across these studies predominantly included severe acquired brain injuries, traumatic brain injuries, and strokes (ischemic and hemorrhagic). Additionally, conditions such as prolonged mechanical ventilation, cardiac surgery complications, and neuromuscular diseases were frequently reported. The observation periods varied significantly, ranging from several months to years. Studies conducted in China [80,83] and Italy [50,51] often spanned multiple years, allowing robust data collection and analysis. In contrast, studies focusing on specific interventions or outcomes, such as those in Spain [43] and the United States [74], typically had shorter observation periods.

### 3.2. Study Design, Research Methods, and Data Collection Tools

Different research designs were utilized to investigate the factors affecting TCT decannulation in critically ill patients and those with acquired brain injury. A randomized controlled trial evaluated two decannulation approaches in critically ill patients on mechanical ventilation, measuring time to decannulation and secondary results [43]. In contrast, three retrospective studies examined data from patients with TCTs to determine factors predicting decannulation. A study concentrated on sABI patients and investigated the link between respiratory parameters and the success of decannulation [44]. A different retrospective study, carried out in China, explored the significance of hypopharyngeal secretion retention, evaluated through the Murray Secretion Scale (MSS), as a predictor for decannulation in severe acquired brain injury (sABI) patients [45]. Ultimately, a significant retrospective research study analyzed the factors influencing decannulation within 6 months for patients with moderate-to-severe neurological injuries [46]. Regarding data collection, retrospective studies employed chart reviews to gather demographic, clinical, and outcome data. The randomized controlled trial gathered data prospectively on decannulation durations, recannulation occurrences, and secondary outcomes such as weaning failure and infections. Clinical evaluations and respiratory function tests, including mean expiratory pressure (MEP), were utilized to determine readiness for decannulation. The MSS was employed to measure the severity of retained hypopharyngeal secretions. The Glasgow Coma Scale (GCS) and National Institutes of Health Stroke Scale (NIHSS) scores were utilized to evaluate the severity of neurological impairment.

### 3.3. Safety and Adverse Events

Thorough oversight of negative events is essential in clinical trials to guarantee patient safety and evaluate the actual risk–benefit balance of treatments. In the analyzed research, negative outcomes were consistently evaluated, categorized, and documented using uniform criteria, frequently adapted to the particular situation of TCT decannulation in seriously ill patients [43,44,45,46]. In general, the studies indicated a low occurrence of significant adverse events (SAEs) directly linked to the decannulation protocols. In the randomized controlled trial conducted by Hernández Martínez et al. [43], the rates of recannulation, which may indicate complications related to the intervention, showed no significant difference between the intervention and control groups. This indicates that the suctioning frequency protocol using continuous high-flow oxygen, although markedly reducing decannulation time, did not jeopardize patient safety. Retrospective studies, despite being constrained by their design, also indicated a small number of intervention-related SAEs. Perin et al. [44] observed that factors leading to non-decannulation comprised excessive pulmonary secretions and infections, yet they did not directly associate these with the decannulation process itself. Song et al. [45] and Wang et al. [46] concentrated on finding predictors of successful decannulation and did not provide details on specific SAEs linked to the decannulation processes. When noted, adverse events were typically mild to moderate in intensity, including problems like slight respiratory distress, brief desaturation, and controllable secretion difficulties. These incidents were generally managed with usual supportive care. Comorbidities, especially severe cardiopulmonary disorders, also adversely affected the success of decannulation. Nevertheless, the possibility of developing more serious complications, like pneumonia or tracheobronchitis, was recognized, highlighting the importance of careful monitoring and customized management approaches.

### 3.4. Predictive Factors for Positive Decannulation Outcomes

Successful decannulation depends on a combination of physiological, functional, and clinical factors, requiring thorough patient evaluation and consistent protocols. Important positive indicators are strong cough force, demonstrated by a peak cough flow (PCF) greater than 100 L/min, and efficient secretion control, reducing aspiration risk [47]. The assessment of swallowing function through Modified Barium Swallow Studies (MBSSs) and respiratory stability, shown by the ability to use speaking valves and tolerate tube capping, is crucial [52]. Neurological conditions, indicated by elevated GCS scores and lack of severe post-hypoxic encephalopathy (PSH), greatly affect outcomes [49]. A younger age consistently appears as a beneficial demographic factor, likely owing to increased physiological reserve [52,53,55]. Timely and comprehensive rehabilitation that includes respiratory, swallowing, and physical therapies enhances patient recovery and readiness for decannulation [62,66,70]. Standardized protocols that include objective metrics such as PCF, MBSSs, and the GCS improve predictive precision and inform clinical decision-making [47,49,59]. The confirmation of no airway obstruction via endoscopic evaluation is essential for effective decannulation [66,77,85]. Moreover, a reduced length of mechanical ventilation, lack of lung infection, and increased alertness are linked to improved results [79,81]. The lack of sepsis and the position of the lesion in the supratentorial region are both favorable indicators [60,61]. On the other hand, an elevated body mass index (BMI), persistent lung conditions, and a greater number of comorbidities serve as unfavorable indicators [74,76]. Multiple other elements are linked to enhanced decannulation success. These consist of a higher Coma Recovery Scale—Revised score upon admission, an oral diet at the time of admission, a shorter period of mechanical ventilation, and a reduced number of complications [48]. Patients experiencing traumatic brain injury or subarachnoid hemorrhage show better outcomes than those suffering from stroke [52]. In a similar vein, elevated Early Functional Abilities scores and diminished Braden and Norton scores, signifying a lower risk of pressure ulcers, are positively associated with successful decannulation [53].

### 3.5. Predictive Factors for Negative Decannulation Outcomes

Negative predictors of decannulation include various physiological, functional, and clinical elements that obstruct successful removal of the TCT tube. Advancing age consistently stands out as a notable negative predictor, indicating reduced physiological reserves and heightened comorbidity burden [75,76,78]. Chronic lung disease affects respiratory function and hinders the process of weaning from mechanical ventilation, ultimately postponing decannulation [78,79]. An elevated BMI is linked to greater reliance on TCT and unsuccessful decannulation because of respiratory issues and airway blockage [47,56,57,88]. Neurological comorbidities, particularly serious brain injuries that lead to low GCS scores or ongoing dysphagia, adversely affect decannulation results [80,82]. Lengthy mechanical ventilation and prolonged stays in the intensive care unit (ICU) suggest a serious condition and a higher chance of complications, postponing decannulation [86,90]. The existence of post-intubation laryngitis, laryngomalacia, or other airway issues detected via endoscopic assessment also obstructs decannulation [64]. Additionally, factors like uncontrolled seizure disorders, repeated subglottic stenosis, and the requirement for repeated laryngotracheal reconstruction lead to failures in decannulation [64]. Complications such as hospital-acquired pneumonia, sepsis, and acute renal injury add to the difficulties of the decannulation process [85,88,89]. Hypoproteinemia and the requirement for passive standing training suggest significant weakness, which adversely impacts decannulation preparedness [65]. Other risk factors comprise diabetes, acute kidney injury, and craniotomy [68]. An increase in ventilator days after TCT and the need for reintubation further decrease the chances of successful decannulation. A high Erasmus GBS Respiratory Insufficiency Score showed a negative correlation with decannulation success, whereas a greater Medical Research Council sum score predicted a positive outcome [75]. Patients with a background of autoimmune diseases or COVID-19 leading to TCT did not exhibit notable differences in decannulation results [76]. Furthermore, the research emphasized that enhancements in swallowing ability were closely linked to decannulation. Patients who no longer needed mechanical ventilation and were able to effectively clear airway secretions showed improved swallowing function after decannulation [73]. The increase in saliva levels of substance P was the sole significant biochemical indicator of enhanced swallowing capacity and effective decannulation, highlighting its possible role as a biomarker in future clinical evaluations [73]. Ultimately, a high Charlson comorbidity index (CCI) score, indicating the presence of several comorbidities, forecasts less favorable decannulation results [54]. Table 3 contains a summary of the included studies.

### 3.6. Impact of Mechanical Ventilation Length on Decannulation Outcomes

Length of mechanical ventilation emerged as a clinically significant factor influencing decannulation outcomes across multiple studies. Patients with shorter ventilator dependency consistently demonstrated higher decannulation success rates and reduced complication profiles [48,79,90]. In contrast, prolonged mechanical ventilation was frequently associated with delayed or failed decannulation, often due to increased comorbid burden, respiratory infections, or critical illness-related neuromuscular weakness [46,73,86]. Notably, Heidler et al. [48] found that reduced ventilator days correlated with successful decannulation in a large cohort of chronically ill patients, while Küchler et al. [79] reported a median time to decannulation of 47 days in patients with subarachnoid hemorrhage, highlighting that even in severe cases, shorter ventilation periods favor recovery. Thomas et al. [90] further observed that a prolonged weaning phase independently predicted lower likelihood of decannulation, reinforcing the need for early mobilization and ventilator liberation when clinically feasible.

**Table 3:** Summary of the included studies.

Author/Location/ Country of the Study	Aim	Study Design/Sample Size/Demographic Data/Diagnosis	Observation/Treatment Period	Data Sources/Follow-Up Duration	Outcome Measures	Main Findings	Factors and Predictors of Decannulation
Hernández Martínez et al. 2020 [43] Location: five ICUs. Country: Spain.	To evaluate the effectiveness of two distinct protocols for TCT decannulation in critically ill adults: one involving capping trials with intermittent high-flow oxygen therapy and the other utilizing suctioning frequency with continuous high-flow oxygen therapy, with a specific emphasis on the duration until decannulation.	Study design: randomized controlled trial. Size: 330 patients (161 in the control group; 169 in the intervention group). Age: mean age of 58.3 ± 15.1 years. Sex: 68.2% male. Diagnosis: critically ill adult patients who underwent their initial TCT while in the ICU. The patients exhibited a range of diagnoses, encompassing medical, trauma, and surgical issues.	From May 2016 to May 2018.	Data sources: data were collected from patient medical records. Follow-up: patients were followed up until hospital discharge or death.	Time for decannulation; decannulation failure, weaning failure, respiratory infections (pneumonia and tracheobronchitis), sepsis, multiorgan failure, durations of stay in the ICU and hospital, ICU readmission, and in-ICU and in-hospital deaths; APACHE II score; Charlson comorbidity index; swallowing test; suctioning frequency; Kaplan–Meier curves.	The research indicated that a protocol utilizing suctioning frequency and continuous high-flow oxygen therapy markedly shortened the duration to decannulation in comparison to capping trials with intermittent high-flow oxygen. Although recannulation rates showed no variation, the frequency group for suctioning had significantly lower rates of weaning failure, pneumonia, and tracheobronchitis. Moreover, this group experienced a notably shorter hospital stay.	The primary factor was the decannulation protocol used: suctioning frequency versus capping trials. Suctioning frequency. Continuous high-flow oxygen therapy. Reduced weaning failure. Reduced respiratory infections.
Perin et al. 2017 [44] Location: hospital and rehabilitation center. Country: Italy.	To determine the factors affecting TCT decannulation in individuals following sABI. In particular, it aimed to identify factors and indicators linked to successful decannulation.	Study design: retrospective observational study. Size: 45 patients. Age: mean age of 67 years, with an interquartile range of 23 (17–84). Sex: 25 men and 20 women. Diagnosis: patients with sABI. The causes of sABI included anoxic brain damage, stroke, and head trauma.	Patients hospitalized from 2011 to January 2014.	Data sources: standardized data collection forms, patient medical records, chest X-rays, and blood test results. Follow-up: the duration of the patients’ hospitalization and rehabilitation.	Decannulation success vs. decannulation failure; GCS, BMI, SpO2, respiratory rate, MIP, and MEP; cough strength and presence (spontaneous or reflex); blood gas analysis; quantification and presentation of pulmonary secretion.	The cause of brain injury significantly affected decannulation success, showing higher rates in patients with head trauma and stroke than in those with anoxic brain injury. A robust and involuntary cough was a crucial indicator of successful decannulation, whereas MEP measurements varied significantly between decannulated and non-decannulated individuals. The primary reasons for decannulation failure were excessive pulmonary secretions and infections, while the GCS score had no significant effect on the results.	Cause of brain injury (stroke; head trauma). Presence and effectiveness of cough (spontaneous and valid). MEP values. Absence of excessive pulmonary secretions. Absence of infections.
Song et al. 2023 [45] Location: a tertiary teaching hospital. Country: not specified.	To investigate the relationship between the severity of hypopharyngeal secretion retention and decannulation results (successful decannulation or extended TCT) in patients with sABI who had a TCT.	Study design: retrospective study. Size: 121 patients. Age: median age of 55.00 years. Sex: 84 (69.4%) males. Diagnosis: sABI. Primary diagnoses included stroke, traumatic brain injury, anoxic encephalopathy, and other conditions like brain tumors or encephalitis.	1 September 2019 to 31 August 2021.	Data sources: electronic medical records and FEES examinations. Follow-up: the follow-up period was during the patient’s stay in the Neurorehabilitation Department. Successful decannulation was defined as no recannulation needed within 72 h of the procedure.	Decannulation outcome, MSS, FEES, and GCS; laryngeal sensation; food/liquid aspiration.	Significant secretion retention (MSS level 3) was independently associated with extended TCT and recognized as the best predictor of decannulation results. Diminished consciousness (GCS ≤ 8) was similarly linked to extended TCT. Although food and liquid aspiration exhibited a relationship with extended TCT in univariate analysis, this connection was not significant in multivariable analysis.	Severity of hypopharyngeal secretion retention (MSS level 3). Consciousness level (GCS score). Laryngeal sensation.
Wang et al. 2022 [46] Location: ICU of the Neurosurgical Department in the First Affiliated Hospital of Nanjing Medical University. Country: China.	To create and confirm a statistical predictive model (nomogram) that measures the likelihood of decannulation within 6 months in patients with moderate or severe neurological injuries receiving TCT treatment.	Study design: retrospective cohort study. Size: 367 patients. Age: mean age of 55.42 ± 13.98 decannulated group, and 59.89 ± 13.06 cannulated group. Sex: males Decannulated: 93 (63.3%) Cannulated: 149 (67.7%) Diagnosis: patients with moderate or severe neurological injury, including traumatic brain injury and stroke (both ischemic and hemorrhagic).	January 2016 to March 2021.	Data sources: retrospective analysis of patient medical records. Follow-up: patients were followed until discharge or until the day of decannulation, with the primary outcome being decannulation within 6 months.	Decannulation within 6 months; GCS; NIHSS; pupillary reactivity; presence of complications (thoracic trauma, inhalation pneumonia, shock, intracranial infection, or epilepsy); early rehabilitation. secondary surgery; TCT indicators; Kaplan–Meier curves.	A nomogram prediction model was created to project decannulation within six months, including age, NIHSS scores, early rehabilitation, shock, and secondary surgery as important predictors. Early rehabilitation was identified as a significant positive influence, whereas advanced age, elevated NIHSS scores, shock, and additional surgeries were associated with a reduced probability of decannulation. The model demonstrated strong discrimination and calibration, suggesting its dependability in clinical settings.	Age (younger age is a positive predictor). NIHSS scores (lower scores are a positive predictor). Early rehabilitation (positive predictor). Shock (negative predictor). Secondary surgery (negative predictor).
Zhou et al. 2022 [47] Location: Beijing Rehabilitation Hospital of Capital Medical University. Country: China.	To examine the effectiveness of decannulation in individuals with extended TCT sent to a rehabilitation hospital, utilizing a standardized multidisciplinary team approach.	Study design: prospective cohort study. Size: 92 patients. Age: mean age of 64.0 years. Sex: 64 (69.6%) males. Diagnosis: patients with prolonged TCT due to various primary diseases, including lung disease, cardiovascular disease, neuromuscular disease, acute brain injury, thoracoabdominal surgery, and cervical spinal cord injury.	January 2019 to September 2021.	Data sources: patient medical records and clinical assessments. Follow-up: 3 months post decannulation.	Success rate of decannulation; decannulation time from referral and reintubation rate; APACHE II score; speaking valve tolerance; upper airway endoscopy; cough strength (PCF and PEF); swallowing assessment (BDT, FEES, and VFSS).	The research showed a high success rate for decannulation (98.2%) employing a standardized multidisciplinary approach, with an average duration of 42.7 days from referral to decannulation. Reintubation three months later was uncommon, and the speaking valve tolerance test was effective in evaluating upper airway openness. Cough strength was identified as a crucial indicator of successful decannulation, whereas swallowing dysfunction did not automatically prevent decannulation.	Clinical stability. Tolerance of the speaking valve. Adequate cough strength (PCF/PEF > 100 L/min). Patency of the upper airway. Multidisciplinary rehabilitation team approach.
Heidler et al. 2018 [48] Location: five ERCs in the Berlin/Brandenburg area. Country: Germany.	To determine elements linked to successful decannulation in patients with tracheostomies receiving ERC.	Study design: uncontrolled experimental study. Size: 831 patients. Age: mean age of 65.4 years. Sex: 68% male. Diagnosis: patients with TCT due to invasive mechanical long-term ventilation, following various critical illnesses including neurological, cardiac, pulmonary, renal, gastrointestinal, oncological, orthopedic, and psychiatric comorbidities.	September 2014 to March 2016.	Data sources: routine medical data collected from the participating clinics. Follow-up: until discharge from the ERC.	Decannulation status at discharge (decannulated vs. non-decannulated); sociodemographic data (age; sex); medical data (type of critical illness, comorbidities, respiratory parameters, and tracheotomy technique); functional assessments (Early Rehabilitation Barthel Index, Bogenhausener Dysphagia Score, and CSR-R); complications (pneumonia, sepsis, laryngeal edema, tracheal stenosis, and tracheomalacia).	Successful decannulation occurred in 57% of patients and was linked to younger age, elevated CRS-R scores, an oral diet upon admission, reduced mechanical ventilation time, and fewer complications. The dilatational TCT method raised the chances of decannulation in comparison to surgical TCT. Conversely, advanced age, extended mechanical ventilation, and complications decreased the likelihood of successful decannulation.	Younger age. Higher CRS-R score at admission. Oral diet at admission. Shorter duration of mechanical ventilation. Fewer complications. Dilatational TCT technique.
Cheng et al. 2019 [49] Location: a tertiary care neurotrauma center. Country: United Kingdom.	To assess the functional and decannulation results of TCT in patients admitted to a HARU.	Study design: observational study. Size: 99 patients. Age: mean age of 52 years (range 17–79). Sex: 60 (60.6%) male, 39 (39.4%) female. Diagnosis: patients with brain injury requiring TCT, including traumatic brain injury, acquired brain injury, intracerebral hemorrhage, tumors, intracerebral infarction, intracranial abscess, and hypoxic brain injury.	A 2-year period.	Data sources: electronic patient notes. Follow-up: until discharge from the HARU.	Decannulation status; GCS, FIM, FAM, PSH, and LOS; discharge destination.	Decannulation was successfully accomplished in 79% of patients, who also showed notable functional advancements according to FIM+FAM scores. Postponed decannulation was frequently linked to a higher secretion burden and recurrent aspiration pneumonia, whereas patients with significant brain injury (low GCS) faced an increased probability of prolonged TCT. PSH extended hospital stays and adversely impacted decannulation, whereas the majority of decannulated patients were sent to intermediate neurorehabilitation units upon discharge.	Higher GCS scores at admission. Absence of severe PSH. Effective management of respiratory and oropharyngeal secretions. Access to a multidisciplinary rehabilitation team.
Reverberi et al. 2019 [50] Location: Neurorehabilitation Unit of the San Sebastiano Community Hospital in Correggio in Reggio Emilia. Country: Italy.	To determine factors that indicate safe decannulation in individuals with sABI and dysphagia and to create a Decannulation Prediction Tool.	Study design: cohort study. Size: 463 patients. Age: mean age of 52.2 years. Sex: 314 (67.8%) males. Diagnosis: patients with sABI and dysphagia, with a TCT cannula on hospital admission. Causes of sABI included anoxia, stroke, trauma, and other causes.	1 November 2003 through 31 December 2016.	Data sources: database of patients admitted to the Neurorehabilitation Unit. Follow-up: until discharge from the Neurorehabilitation Unit.	Safe decannulation before discharge; demographics; brain lesion date of onset and pathogenesis; presence of vegetative status or minimal consciousness state; saliva aspiration (blue dye test); voluntary and reflex cough; severity of dysphagia (FOIS).	Prior to discharge, 73% of patients were safely decannulated, with younger age, no vegetative state, saliva aspiration, traumatic brain injury, and the presence of voluntary and reflexive cough recognized as significant predictors. The decannulation prediction tool, created with these factors, showed strong discriminative capability in forecasting decannulation success. Furthermore, patients demonstrated notable enhancement in FOIS scores from their admission until discharge.	Younger age. Absence of vegetative status. Absence of saliva aspiration. Traumatic brain injury. Presence of voluntary and reflex cough.
Zivi et al. 2018 [51] Location: a neurorehabilitation unit. Country: Italy.	To assess if an early neurorehabilitation protocol shortens the time to decannulation in TCT patients with sABI.	Study design: retrospective cohort study. Size: 66 patients. Age: mean age of 61.4 ± 15.7 years in the early rehabilitation group and 58.2 ± 14.7 years in the delayed rehabilitation group. Sex: early rehabilitation group (N = 40)—25 males and 15 females; delayed rehabilitation group (N = 26)—22 males and 4 females. Diagnosis: sABI requiring TCT, including traumatic, ischemic, hemorrhagic, anoxic, and other brain injuries.	3 years.	Data sources: retrospective evaluation of patient medical records. Follow-up: until discharge from the neurorehabilitation unit.	TCT duration (time to decannulation); ICU length of stay, time to decannulation in the neurorehabilitation unit, re-cannulation rate, GCS score, CRS-R score, and Levels of Cognitive Functioning score; Kaplan–Meier curves; Cox proportional hazards model.	Patients who underwent early neurorehabilitation in the ICU experienced a notably shorter TCT duration and ICU stay than those who began rehabilitation in the neurorehabilitation unit. Furthermore, early rehabilitation exhibited a tendency for quicker decannulation and improved neurological condition, especially in individuals who participated in stepping verticalization sessions in the ICU. Nonetheless, the duration until decannulation in the neurorehabilitation unit was similar for both groups.	Early neurorehabilitation in the ICU (positive predictor). Shorter bed rest. Stepping verticalization sessions in the ICU (trend towards positive influence).
Mortensen et al. 2020 [52] Location: Hammel Neurorehabilitation Centre and University Research Clinic. Country: Denmark	To create a predictive model and an online resource for estimating the duration until decannulation in individuals with ABI.	Study Design: Retrospective Cohort Study. Size: 574 tracheostomized patients. Age: median age of 54 years (range 3–85 years). Sex: 208 (36%) female. Diagnosis: ABI, including stroke (ischemic or hemorrhagic), TBI, subarachnoid hemorrhage, encephalopathic brain injury, and other injuries.	March 2011 to the end of 2018.	Data sources: electronic medical records. Follow-up: until decannulation or discharge from the rehabilitation hospital.	Time to the first attempt of decannulation from any type of TCT tube; time to decannulation from a cuffed tube specifically; EFA score.	A predictive model and web-based tool were created to estimate the decannulation duration. The most significant predictors were age and functional status (EFA score and swallowing ability), with a younger age and elevated EFA scores linked to a higher probability of decannulation. In total, 72% of patients underwent decannulation, and the tool offers probability estimates along with confidence intervals.	Younger age (positive predictor). Higher Early Functional Abilities (EFA) score (positive predictor). Presence of swallowing function (positive predictor). Diagnosis of TBI or subarachnoid hemorrhage (positive predictor compared to stroke). Shorter time between injury and admission to rehabilitation.
Zengin et al. 2024 [53] Location: PCC of a research and training hospital. Country: Turkey.	To examine factors affecting TCT decannulation in patients admitted to a PCC.	Study design: retrospective study. Size: 102 patients. Age: mean age of 52.10 ± 20.62 years (decannulated) and 61.48 ± 18.07 years (non-decannulated). Sex: male—70.7% (decannulated) and 68.9% (non-decannulated); female—29.3% (decannulated) and 31.1% (non-decannulated). Diagnosis: patients with TCTs, often referred from ICUs, with various underlying conditions including trauma, cerebrovascular events, oncological diagnoses, chronic obstructive pulmonary disease, aspiration pneumonia, and cardiovascular diseases.	2017 to 2022.	Data sources: retrospective analysis of patient information from hospital records. Follow-up: during hospitalization in the PCC.	Decannulation status; demographic data; diagnoses and comorbidities; nutritional status; length of hospital stay; pressure ulcer status (Norton and Braden scales); discharge status; blood gas analysis.	Younger patients who were decannulated had a longer duration of hospital stay compared to those who were not decannulated. A reduced risk of pressure ulcers (elevated Braden and Norton scores) correlated with increased decannulation rates, whereas gender, malnutrition, oxygen therapy, and tube feeding did not demonstrate significant effects. Blood gas measurements were taken, and a focus on multidisciplinary care, encompassing respiratory and swallowing therapy, was highlighted.	Younger age (positive predictor). Lower risk of pressure ulcers (higher Braden and Norton scores) (positive predictor). Multidisciplinary care. Effective respiratory and swallowing therapy. Absence of exacerbating comorbidities such as advanced heart failure, cancer, or Alzheimer’s disease.
Higashi et al. 2019 [54] Location: Yokohama City University School of Medicine. Country: Japan.	To investigate the related factors for TCT and decannulation in older patients with traumatic CSCI.	Study design: retrospective study. Size: 65 patients. Age: ranging from 60 to 94 years, with a mean age of 72.8 ± 8.3 years. Sex: 48 males (74%) and 17 females (26%). Diagnosis: traumatic CSCI.	January 2010 to June 2017.	Data sources: patient medical records from the Outpatient Department and reports from hospitals where patients were admitted. Follow-up: one year after injury.	Time for decannulation; mortality (in-hospital and within one year); AIS, NLI, and CCI; ICU length of stay; intubation at arrival; radiological findings.	Severe AIS at the time of injury, significant fractures, surgical intervention, and initial intubation were critical elements that resulted in TCT, whereas an elevated CCI was associated with failure to decannulate. The total decannulation rate stood at 30%, but for those who did not succeed in decannulation, mortality soared to 35%.	High CCI (negative predictor). Severe AIS (positive predictor). Major fracture or dislocation (positive predictor). Operative treatment (positive predictor). Intubation at arrival (positive predictor).
Park et al. 2018 [55] Location: rehabilitation center of a university hospital, specifically a regional cerebrovascular center. Country: South Korea.	To illustrate alterations in swallowing and voluntary coughing in stroke patients prior to and following TCT decannulation.	Study design: observational study. Size: 77 patients. Age: decannulated group—mean age 50.6 ± 11.0 years; non-decannulated group—mean age 63.7 ± 10.1 years. Sex: decannulated group—20 males and 15 females; non-decannulated group—23 males and 19 females. Diagnosis: subacute stroke patients.	March 2015 to December 2016.	Data sources: patient medical records and functional evaluations conducted within the rehabilitation center. Follow-up: up to 6 months post stroke.	FDS, PAS, PCF, K-MMSE, K-MBI, and VFSS.	Post TCT decannulation, cough ability markedly enhanced, whereas swallowing function displayed no immediate alterations. Nevertheless, patients who were decannulated showed improved overall functional gains in swallowing, coughing, cognition, and daily activities when compared to those who continued to be tracheostomized. An earlier age at stroke onset raised the chances of successful decannulation, whereas the initial stroke features did not affect the outcome.	Younger age at stroke onset (positive predictor). Improvement of swallowing and cough functions (positive predictor). Improvement of ADL and cognitive functions (positive predictor). Multidisciplinary approach.
Enrichi et al. 2017 [56] Location: Neurorehabilitation Department of Fondazione Ospedale San Camillo in Venice. Country: Italy.	To examine the key and objective factors for TCT tube removal (decannulation) in individuals with post-acute ABI.	Study design: cross sectional study. Size: 74 participants. Age: mean age 51.5 ± 16.8 years. Sex: 32 females. Diagnosis: ABI with TCT cannula.	2 July 2015, to 31 July 2016.	Data sources: clinical assessments, instrumental evaluations, and patient records from the Neurorehabilitation Department. Follow-up: 48 h after TCT tube removal.	TCT tube capping tolerance; assessment of dysphagia (swallowing difficulties); voluntary cough (maximum expiratory pressure, PCF); reflex cough (citric acid nebulization); swallowing instrumental assessment (fibro-endoscopic evaluation, penetration aspiration scale); blue dye test; number of tracheal suctions; endoscopic assessment of airway patency; oxygen saturation; level of consciousness (GCS).	A mix of TCT tube capping, swallowing evaluations, tracheal suction frequency, and the blue dye test exhibited the greatest sensitivity and specificity for forecasting successful decannulation. The highest predictive accuracy was obtained by merging an “airway patency cluster” (tube capping and endoscopic evaluation) with a “dysphagia cluster” (blue dye assessment and swallowing test). Although voluntary cough evaluation revealed high sensitivity but low specificity, probably because of cognitive impairments in ABI patients, reflex cough testing exhibited high specificity.	Tolerance of TCT tube capping. Patent airway (endoscopic assessment). Absence of aspiration (swallowing instrumental assessment, blue dye test). Effective secretion management (low number of tracheal suctions).
Kang et al. 2016 [57] Location: Department of Rehabilitation Medicine at Gangnam Severance Hospital. Country: South Korea.	To assess the clinical significance of PCF measured with an external control device replacing glottic function regarding TCT decannulation in individuals with neuromuscular disorders or cervical spinal cord injuries.	Study design: retrospective study. Size: 16 patients. Age: average age of 43.9 years (range 19–76 years). Sex: 12 males and 4 females. Diagnosis: neuromuscular diseases (Guillain–Barre syndrome, Duchenne muscular dystrophy, Kennedy disease, progressive muscular dystrophy, and polymyositis) and CSCI.	February 2009 to September 2014.	Data sources: patient medical records and measured PCF values. Follow-up: during the research period, patients were monitored for respiratory complications and rehospitalization.	UPCF, APCF, and APCF-ECD.	The external control device effectively raised PCF in patients who failed to satisfy typical decannulation criteria, enabling all 16 patients to be safely decannulated. Following decannulation, APCF was markedly greater than APCF-ECD prior to the intervention. No significant respiratory issues were noted after decannulation.	APCF-ECD reaching 160 L/min or more. Tolerance of TCT tube capping. Absence of anatomical airway abnormalities. Ability to transition to non-invasive ventilation. Absence of severe swallowing dysfunction.
Ge et al. 2024 [58] Location: Beijing Rehabilitation Hospital of Capital Medical University. Country: China.	To determine whether a CFSV exceeding 100 L/min, assessed with a speaking valve fitted, could serve as a dependable standard for successful TCT decannulation in patients experiencing prolonged TCT from diverse underlying conditions.	Study design: prospective cohort study. Size: 191 patients. Age: mean age of 63.38 ± 15.94 years for the decannulated group and 64.67 ± 11.45 for the non-decannulated group. Sex: 135 males and 58 females. Diagnosis: patients with prolonged TCT due to various primary diseases, categorized as pulmonary disease, acute brain injury, VPF, thoracoabdominal surgery, or multiorgan failure.	January 2019 to September 2022.	Data sources: patient medical records, CFSV measurements, and clinical assessments. Follow-up: 6 months.	CFSV, VPF, PEF, APACHE II, and GCS.	A CFSV measurement exceeding 100 L/min demonstrated a dependable sign of effective decannulation, which showed a high success rate and low requirement for reinsertion. Pulmonary rehabilitation, especially methods for enhancing cough, played a crucial role in advancing CFSV and aiding decannulation. Nonetheless, CFSVs and air leak degrees fluctuated according to the patient’s main illness.	CFSV greater than 100 L/min. Tolerance to the speaking valve. Clinical stability. Effective pulmonary rehabilitation.
Mannini et al. 2021 [59] Location: IRCCS Fondazione Don Carlo Gnocchi in Florence. Country: Italy.	To explore factors influencing the likelihood and timing of TCT decannulation in patients with sABI who are admitted to intensive rehabilitation units and to create machine learning models for forecasting these results.	Study design: retrospective study. Size: 327 patients. Age: median age of 67.18 years. Sex: 210 males and 117 females. Diagnosis: sABI.	1 August 2012 to 31 January 2019.	Data sources: demographic data, clinical and functional scales, vital support information, clinical evaluations, and instrumental data (electromyography, bronchofibroscopy) from patient records. Follow-up: the study used the data collected during the patients stay in the intensive rehabilitation unit, and until discharge.	CRS-R, DRS, LCF, GCS, FOIS, and FIM; electromyography; bronchofibroscopy.	The level of consciousness, functional assessments, and whether a feeding tube was in place were significant predictors of the likelihood of decannulation, while variables such as age, body mass index, the cause of brain injury, and mechanical ventilation impacted the timing of decannulation. Machine learning models, such as Weighted Posteriors Voting and AdaBoost SVR, demonstrated encouraging precision in forecasting both probability (84.8%) and timing (median error of 25.7 days). These results underscore the promise of artificial intelligence-based methods in enhancing decannulation choices.	Higher consciousness level (CRS-R, GCS). Better functional status (FOIS, FIM). Presence of nasogastric intubation. Absence of PEG. Younger age. Lower BMI. Traumatic brain injury etiology. Absence of mechanical ventilation need.
Hakiki et al. 2020 [60] Location: Intensive Rehabilitation Unit of the Don Carlo Gnocchi Foundation in Florence. Country: Italy.	To examine the frequency and timing of TCT decannulation in individuals with sABI within a rehabilitation environment, and to assess the impact of clinical traits on the decannulation process.	Study design: uncontrolled experimental study. Size: 351 patients. Age: mean age of 64.1 ± 15.5 years. Sex: 125 females. Diagnosis: sABI.	1 August 2012 to 31 January 2019.	Data sources: patient database; medical records. Follow-up: until discharge from the intensive rehabilitation units or death.	Patient database, clinical and functional assessments, CRS-R, GCS, fibrobronchoscopy results, and FOIS.	Decannulation was achieved in 54.1% of patients, with improved results associated with the lack of respiratory infections, tracheal changes, and sepsis. Elevated CRS-R and GCS scores, coupled with an improved clinical condition (MCS, E-MCS) upon admission, were also significant indicators of success. Though pulmonary infections, tracheal problems, and sepsis postponed decannulation, patients in MCS and E-MCS accomplished it faster than those in UWS.	Absence of respiratory infections. Absence of tracheal alterations. Absence of sepsis. Higher CRS-R score. Higher GCS score. MCS. E-MCS.
Schneider et al. 2017 [61] Location: Neurological, surgical, and anesthesiological ICUs of the University Hospital of Dresden. Country: Germany.	To examine decannulation rates and factors influencing decannulation in individuals with severe stroke who received TCT and to evaluate their functional outcomes.	Study design: observational study. Size: 53 patients. Age: mean age of 61.4 years. Sex: 31 males and 22 females. Diagnosis: intracerebral hemorrhage, ischemic stroke, or subarachnoid hemorrhage.	May 2014 to April 2015.	Data sources: electronic case report forms, patient medical records, structured telephone interviews with patients, relatives, or caregivers. Follow-up: 12 months after TCT.	mRS, Barthel index, NIHSS, GCS, ACS; brain imaging data (CT and MRI).	Decannulation was achieved in 35.8% of patients in less than 12 months, with a younger age and lack of sepsis serving as independent predictors of successful outcomes. Patients who were decannulated demonstrated improved functional results at the 12-month point, and infratentorial lesions were notably rarer in these individuals. These results emphasize important elements affecting successful decannulation.	Younger age. Absence of sepsis. Supratentorial lesion location.
Hakiki et al. 2022 [62] Location: Intensive Rehabilitation Unit of the IRCCS Don Gnocchi Foundation of Florence. Country: Italy.	To examine the correlation between TCT decannulation and enhancement in responsiveness in individuals with sABI and DoC throughout intensive rehabilitation.	Study design: retrospective cohort study. Size: 236 patients. Age: median age of 67 years. Sex: 86 females. Diagnosis: sABI with DoC, including UWS, and MCS.	1 August 2012 to 31 January 2019.	Data sources: patient database and medical records. Follow-up: until discharge from the Intensive Rehabilitation Unit, with a final assessment in July 2019.	CRS-R; decannulation status; improvement in responsiveness, defined as transition from UWS to MCS or E-MCS, or from MCS to E-MCS.	A significant connection was noted between decannulation and enhanced responsiveness at discharge in both UWS and MCS patient groups. Decannulation was accomplished in several patients originally admitted with UWS, indicating that it significantly contributes to improving levels of consciousness. These results emphasize decannulation as a crucial element linked to enhanced levels of awareness.	Decannulation was the strongest predictor of improvement of responsiveness. In the MCS group, shorter post-onset time was associated with improvement of responsiveness.
Heidler et al. 2014 [63] Location: a German rehabilitation clinic. Country: Germany.	To examine factors influencing successful TCT decannulation in patients moved from ICUs to a rehabilitation center.	Study Design: Retrospective Study. Size: 150 patients. Age: Mean age 65.5 years. Sex: 66% male. Diagnosis: patients tracheotomized in ICUs for mechanical ventilation due to various conditions (neurological, cardiological, respiratory, gastrointestinal).	Data collected from August 2011 to August 2012 (13 months).	Data sources: regularly gathered patient information (age, sex, diagnoses, complications, comorbidities, body mass index, TCT method, length of mechanical ventilation, dependence on nursing care, alertness/responsiveness, and endoscopic tracheoscopy results). Follow-up: until discharge from the rehabilitation clinic, with assessment of decannulation status two weeks post removal.	Decannulation status (decannulated vs. non-decannulated); CRS for alertness/responsiveness; Early Rehabilitation Barthel Index; aspiration scale; endoscopic tracheoscopy findings.	A total of 68.7% of patients underwent successful decannulation, with complications arising during the procedure being the best predictor of failure. Increased alertness and responsiveness upon admission, indicated by the CRS score, notably raised the chances of success. Conversely, being male and having more comorbidities were linked to reduced decannulation rates. Furthermore, individuals who had dilatational tracheotomies were more likely to achieve successful decannulation.	Complications during decannulation. Alertness/responsiveness (CRS score). Gender (female). Number of comorbidities (lower). TCT technique (dilatational).
Schweiger et al. 2020 [64] Location: four high complexity healthcare referral centers. Country: Brazil	To determine the predictors of TCT decannulation in children who had TCT.	Study design: retrospective cohort study. Size: 160 patients. Age: median age at TCT of 6.96 months. Sex: 58.1% male. Diagnosis: pediatric patients (under 18 years) who underwent TCT for various indications, including post-intubation laryngitis, congenital laryngeal stenosis, prolonged mechanical ventilation, laryngomalacia, and others.	January 2013 to December 2015.	Data sources: medical records. Follow-up: from the time of TCT until decannulation or the last medical visit.	TCT decannulation status; Cox proportional hazards regression model.	The rate of TCT decannulation was 22.5%, with a higher age at TCT and post-intubation laryngitis as factors boosting the chances of success. Conversely, neurological and pulmonary comorbidities, combined with a greater total of comorbidities, greatly decreased the likelihood of decannulation. A significant incidence of complications, both early and late, was noted, and the total mortality rate reached 18.1%.	Older age at TCT. Post-intubation laryngitis (and a trend for laryngomalacia) as indication. Neurological comorbidities (negative predictor). Pulmonary comorbidities (negative predictor). The number of cumulative comorbidities (negative predictor).
Zeng et al. 2024 [65] Location: ten hospitals in mainland China. Country: China.	To investigate the factors influencing TCT decannulation results in patients with a PVS and to create a nomogram for forecasting decannulation success.	study design: retrospective multicenter study. Size: 872 individuals. Age: median age of 52 years. Sex: 471 males (54%) and 401 females (46%). Diagnosis: PVS requiring TCT.	Data collection occurred in 2022.	Data sources: retrospective medical records. Follow up: not specified.	TCT decannulation success; nomogram development and validation; ROC curves; calibration curves; DCA.	Ten factors were recognized as linked to successful decannulation, and a nomogram was created with validation conducted both internally and externally. Factors that contributed to decannulation failure included extended TCT tube duration, lung infection, low protein levels, absence of passive standing practice, atypical swallowing reflex, use of mechanical ventilation, prolonged ICU admission, and advanced age. Conversely, favorable elements for successful decannulation comprised oral feeding, passive standing exercises, having a personal caregiver, and a reduced ICU duration.	Duration of TCT tube placement. Pulmonary infection. Hypoproteinemia. Passive standing training. Swallowing reflex. Mechanical ventilation. ICU duration. Age. Peroral feeding. Private caregiver.
Bishnoi et al. 2022 [66] Location: Command Hospital Bangalore. Country: India.	To prospectively outline the factors affecting TCT decannulation in a group of patients.	Study design: prospective descriptive study. Size: 50 TCT patients. Age: 18–85 years. Sex: 40 males and 10 females. Diagnosis: Patients who underwent emergency or planned TCT due to difficulties with extended mechanical intubation, stridor, or other respiratory issues. Registered patients with severe head injuries, head trauma linked to multiple injuries, meningitis/meningoencephalitis, strokes, and a range of other ailments.	January 2019 to April 2020.	Data sources: patient medical records, clinical assessments, FOL, tracheal aspirate swabs, and chest X-rays. Follow-up: the research centered on the decannulation procedure and the immediate post-decannulation phase, particularly highlighting the initial 24–48 h. It was mentioned that there was insufficient follow-up following a period of 6 months.	Decannulation success or failure; assessment of cough effectiveness and swallowing and gag reflexes; FOL; tracheal aspirate microbiological evaluation; chest X-ray; penetration aspiration scale; SpO2 levels.	The rate of decannulation failure was 14%, with contributing factors comprising weak or absent swallow, cough, and gag reflexes, desaturation events, and excessive mucus. Successful secretion management was found to be essential, while stridor also contributed to failure. The significance of a uniform decannulation protocol to enhance results was emphasized.	Cough effectiveness. Swallowing and gag reflexes. Secretion management. Airway patency (absence of tracheal stenosis). SpO2 levels. Absence of pulmonary pathology.
Leto et al. 2021 [67] Location: Institute S. Anna, Crotone. Country: Italy.	To confirm and possibly enhance the DecaPreT model, a forecasting instrument for successful TCT decannulation in individuals with ABI.	Study design: retrospective study. Size: 273 ABI patients. Age: mean age at injury of 53.01 ± 17.75 years. Sex: 93 females (34.07%). Diagnosis: ABI with GCS ≤ 8 at ICU discharge.	January 2016 to December 2020.	Data sources: patient medical records, including ICU records and neurorehabilitation unit assessments. Follow-up: the endpoint was defined as safe decannulation, which means decannulation that is not followed by aspiration or the necessity for re-TCT within 48–96 h.	Safe TCT decannulation; CRS-R; FOIS; DecaPreT score; statistical analysis included likelihood ratio tests, Brier score, area under the ROC curve, calibration plots, and reclassification metrics.	Prior to discharge, 61.5% of patients had been successfully decannulated. The initial DecaPreT model was updated by integrating variables like age, CRS-R, and ICU length of stay, leading to enhanced predictive accuracy. The full model, which accounted for ICU length of stay, exhibited the greatest accuracy and discrimination, whereas the continuous model, which included age and CRS-R, displayed the best calibration.	Age at injury. CRS-R score at admission. Length of stay in ICU. DecaPreT original scores, including brainstem injury, saliva aspiration, vegetative state, coughing score, and pathogenesis of brain lesion.
Jenkins et al. 2020 [68] Location: University of Maryland Medical Center, Baltimore, MD, and its affiliated rehabilitation facility. Country: USA.	To determine clinical factors linked to TCT decannulation in patients suffering from severe TBI who need TCT.	Study design: retrospective study. Size: 79 patients. Age: mean age of 45 years. Sex: 78% male. Diagnosis: severe TBI with GCS ≤ 8 or loss of consciousness >24 h, requiring ICU admission for ≥72 h and TCT.	1 January 2014 to 31 December 2014.	Data sources: hospital trauma registry and patient medical records. Follow-up: during their time at a connected rehabilitation center and up to the moment of decannulation.	Time to TCT decannulation; Cox Proportional Hazards model; GCS score; Abbreviated Injury Scale–Head; ISS; Marshall and Rotterdam scores; lung injury score; CPC score; Rancho Los Amigos Levels of Cognitive Functioning Scale score.	Seventy-five percent of patients had their cannulas removed within 90 days. Nonetheless, diabetes, acute kidney injury, and craniotomy were associated with a decreased probability of decannulation prior to TCT, whereas reintubation, aspiration, extended ventilator use, and acute kidney injury diminished the odds of decannulation at the time of hospital discharge. Conversely, advanced age was linked to an increased chance of successful decannulation. Furthermore, approximately one in ten patients who had TCT experienced subglottic stenosis and issues with speech.	Diabetes (negative predictor). Acute kidney injury (negative predictor). Craniotomy (negative predictor). Reintubation (negative predictor). Aspiration (negative predictor). Increased ventilator days after TCT (negative predictor). Age (positive predictor).
Lind et al. 2017 [69] Location: pediatric sleep center in a tertiary children’s hospital. Country: USA.	To determine if functional status and comorbid conditions correlate with the ability to decannulate children who met decannulation criteria based on cPSG.	Study design: retrospective study. Size: 104 unique patients (139 sleep studies). Age: median age of 4 years. Sex: not reported. Diagnosis: children who underwent evaluation with cPSG for possible TCT decannulation. Common comorbid conditions included cerebral palsy, BPD, chronic lung disease, congenital or acquired neuromuscular diseases, congenital heart disease, and a history of organ transplantation.	February 2005 to September 2015.	Data sources: patient charts and electronic medical records. Follow-up: one year after cPSG predicted readiness for TCT decannulation.	Continued presence of a TCT at 1 year after cPSG. AHI. ETCO2 levels; functional status assessment (locomotion, self-care, and communication); Mann–Whitney U tests and Fisher exact tests; Spearman rank correlation coefficients; exact Cochran–Armitage trend tests.	At the end of one year, 79.8% of patients had been successfully decannulated. The functional status and accompanying conditions did not independently forecast decannulation success in children who fulfilled the criteria based on cPSG. Nonetheless, individuals with three or more chronic illnesses and a functional status under 7 were less likely to achieve successful decannulation. No notable patterns were seen in decannulation suggestions as functional scores rose.	Number of comorbid conditions (3 or more, negative predictor in conjunction with low functional status). Total functional status score (<7, negative predictor in conjunction with a high number of comorbid conditions).
Draghi et al. 2024 [70] Location: Istituto di Ricovero e Cura a Carattere Scientifico (IRCCS), Fondazione Don Gnocchi of Florence. Country: Italy.	To evaluate the state of consciousness upon achieving decannulation in patients with pDoC and to identify the timing for decannulation.	Study design: uncontrolled experimental study. Size: 144 patients. Age: median age of 69 years. Sex: 56 women (39.7%). Diagnosis: patients with pDoC following sABI.	June 2020 to September 2022.	Data sources: patient medical records, EEG results, and clinical evaluations. Follow-up: during the intensive rehabilitation unit stay, up to discharge.	Consciousness state at decannulation (CRS-R); decannulation timing; EEG; Cumulative Illness Rating Scale; Kaplan–Meier method; Log-rank test; Mann–Whitney U test; chi-square test; linear regression models.	Patients who were decannulated exhibited a higher level of consciousness upon admission and had a greater likelihood of attaining improved responsiveness more quickly. The timing of decannulation was associated with both the initial IR and the existence of normal voltage on the baseline EEG. Clinically observable indications of consciousness consistently occurred before decannulation, with 97.3% of patients displaying progress prior to the procedure. Importantly, following decannulation, no patient continued in an unresponsive wakefulness syndrome (UWS/VS) state.	Higher consciousness level at admission (positive predictor). Improved responsiveness during IRU stay (positive predictor). Shorter time post onset (positive predictor). Normal voltage on EEG (positive predictor). Absence of pulmonary infections (positive predictor). Shorter time to the first IR (positive predictor).
Kim et al. 2015 [71] Location: Myongji Hospital Rehabilitation Medicine Department. Country: South Korea.	To examine the alterations in swallowing abilities, particularly concentrating on laryngeal lifting, pharyngeal transit duration, residual pharyngeal content post-swallowing, upper esophageal diameter, and aspiration of semisolid foods, prior to and following decannulation in individuals with brain injuries.	Study design: retrospective study. Size: 17 patients. Age: average age of 58.88 ± 14.05 years. Sex: 12 male and 5 female patients. Diagnosis: brain injuries from cerebral hemorrhage and/or infarction, TBI, or brain hypoxia, who underwent decannulation.	Patients admitted between 1 May 2012 and 28 February 2014.	Data sources: VFSS and patient medical records. Follow-up: VFSS performed within 1 month prior to decannulation and again within 1 month after the procedure.	Laryngeal elevation (x- and y-axis); pharyngeal transit time; post-swallow pharyngeal remnant; upper esophageal width; semisolid aspiration (penetration–aspiration scale—PAS); Wilcoxon signed-rank tests.	Statistically significant variations were noted in post-swallow pharyngeal remnant and upper esophageal width after decannulation. Nevertheless, no substantial differences were observed in laryngeal elevation, pharyngeal transit duration, or semisolid aspiration, indicating that these aspects were not significantly influenced by the decannulation process.	This research concentrated on how decannulation impacts swallowing function, rather than on identifying predictors of the decannulation process. The study suggests that patients who do not need a ventilator anymore and can physiologically expectorate sputum will see improvements in swallowing function after decannulation. Dressing the decannulation site might positively influence swallowing function.
Ghiani et al. 2022 [72] Location: Schillerhoehe Lung Clinic, Gerlingen. Country: Germany.	To investigate the factors and indicators contributing to decannulation failure in patients who received extended mechanical ventilation and weaning at a specialized facility.	Study design: retrospective study. Size: 532 patients. Age: median age of 70 years. Sex: 62.4% male. Diagnosis: patients sent for the process of weaning from TCT ventilation after extended mechanical ventilation.	June 2013 to January 2021.	Data sources: electronic medical records and charting systems from the hospitals. Follow-up: until hospital discharge.	Decannulation success and failure rates; causes of decannulation failure; demographics, clinical characteristics, and comorbidities. APACHE-II; Charlson comorbidity index; albumin levels; modified Evan’s blue dye test; FEES; penetration–aspiration scale; multivariable binary logistic regression analysis; Hosmer and Lemeshow test; Nagelkerke R2.	Decannulation failure happened in 41% of patients, mainly because of significant dysphagia and prolonged reliance on ventilators. The main risk factors consisted of age, BMI, APACHE-II score, prior domiciliary non-invasive ventilation, percutaneous TCT, neuromuscular disorders, and overall length of mechanical ventilation. Nonetheless, non-invasive ventilation was effectively employed to assist with decannulation in certain patients who faced weaning failure.	Age, body mass index, APACHE-II scores, pre-existing domiciliary non-invasive ventilation, percutaneous TCTs, neuromuscular diseases, and total duration of (invasive) mechanical ventilation were independently related to decannulation failure. Severe dysphagia and long-term ventilator dependence were the primary causes of decannulation failure. Factors that affected those categories were also analyzed.
Muhle et al. 2017 [73] Location: Neurological ICU of the University Hospital Muenster. Country: Germany.	To analyze the effect of PES on substance P saliva levels and its correlation with decannulation success in stroke patients with dysphagia who have undergone TCT.	Study design: prospective single-center study. Size: 23 patients. Age: mean age of 64.43. Sex: 11 females and 12 males. Diagnosis: Ischemic or hemorrhagic stroke patients with severe and persisting dysphagia, requiring TCT.	October 2014 to August 2015.	Data sources: FEES, saliva samples, and patient medical records. Follow-up: during hospitalization, until decannulation or discharge.	Decannulation success; substance P concentration in saliva; FEES; NIHSS; TOAST criteria.	A rise in SP saliva levels following PES was closely associated with enhanced swallowing ability and elevated decannulation success rates, serving as the sole independent predictor of successful decannulation. Ongoing PES treatment cycles further increased SP levels, resulting in improved results for certain patients. These results indicate that PES might have a beneficial effect on both central and peripheral components of the swallowing network.	The only significant predictor of enhanced swallowing function and successful decannulation was the rise in saliva concentration of substance P. The research indicated that factors such as age, gender, prior dysphagia/comorbid conditions, NIH-SS, and length of artificial ventilation were not key predictors of successful decannulation in this investigation.
Meenan et al. 2021 [74] Location: two acute rehabilitation centers associated with the University of Maryland. Country: United States.	To determine risk factors for the formation of laryngeal lesions that prevent TCT decannulation in individuals who have undergone intubation and TCT.	Study design: retrospective study. Size: 371 patients. Age: average age of 52.2 ± 17.8 years. Sex: 234 male patients (63.1%) and 137 female patients (36.9%). Diagnosis: patients with TCTs following intubation.	April 2016 to November 2019.	Data sources: patient medical records and endoscopic airway evaluation reports. Follow-up: not specified.	Presence of laryngeal lesions precluding decannulation; demographics, comorbidities, and intubation-related factors; flexible fiberoptic laryngoscopy and/or tracheoscopy.	Laryngeal lesions related to intubation obstructed decannulation in 13.2% of patients, with PGS identified as the primary cause. An elevated BMI (≥25 kg/m^2^) notably raised the likelihood of forming these lesions, whereas a smoking history seemed to lower the chances. Nonetheless, 77.5% of patients with obstructive lesions were effectively decannulated following medical and/or surgical intervention.	BMI ≥ 25 kg/m^2^ was a significant risk factor for laryngeal lesions precluding decannulation. Former smoking history was shown to decrease the odds of developing laryngeal lesions. Age, sex, race, history of diabetes mellitus, duration of intubation, number of intubations per person, and size of the endoscopic endotracheal tube were not associated with increased risk of precluding lesions.
Schröder et al. 2019 [75] Location: Department of Neurology at the University Hospital Muenster. Country: Germany.	To examine dysphagia in GBS patients, emphasizing its effects on intubation and decannulation.	Study design: retrospective study. Size: 88 patients. Age: mean age of 57.0 ± 16.8 years. Sex: 56 male patients (63.6%). Diagnosis: GBS.	April 2005 to January 2016.	Data sources: patient medical records, results of cerebrospinal fluid analysis, results of serological screening for preceding infections, and FEES. Follow-up: during hospital stay, until discharge.	Intubation requirement; decannulation success; dysphagia screening (water swallow test) FEES; Medical Research Council sum score; EGRIS; FOIS.	Weakness in respiratory muscles, instead of dysphagia, was the primary cause for intubation, while profound dysphagia notably postponed decannulation following respiratory weaning. Numerous patients suffering from ongoing dysphagia also showed significant laryngeal sensory impairments. The EGRIS was found to be a significant predictor of the necessity for intubation and postponed decannulation, while also being independently linked to the success of decannulation.	EGRIS was inversely correlated with decannulation success. Severe dysphagia, particularly sensory deficits, delayed decannulation. Facial/bulbar weakness was significantly more present in the non-decannulated group. A higher Medical Research Council sum score was correlated with decannulation success. The duration of ventilation was longer in the non-decannulated group. Age, BMI scores, bulbar/facial weakness, a pathological WST, and the duration of ventilation were not independently correlated to decannulation success in the binary logistic regression analysis.
Wehbi et al. 2024 [76] Location: Voice and Swallowing Center at the University of Arizona. Country: United States.	To assess the results of endoscopic treatment of SGS preventing decannulation and to determine factors that forecast successful decannulation.	Study design: retrospective study. Size: 22 patients. Age: mean age of 57.5 years. Sex: 13 (59.1%) females and 9 (40.9%) males. Diagnosis: SGS precluding decannulation.	2018 to 2023.	Data sources: medical records. Follow-up: the study covers the period that the patients were treated.	TCT decannulation; patient demographics (age, gender, and BMI); clinical variables (comorbidities, pulmonary disease, and COVID-19 as cause of TCT); stenosis characteristics (grade, location, granulation tissue, tracheomalacia, EDAC, multilevel stenosis, and posterior glottic stenosis); number of endoscopic treatments.	Endoscopic intervention resulted in successful decannulation for 40.9% of the patients, with BMI scores and age identified as important negative predictors of success. Methods like CO_2_ laser debridement, balloon dilation, and intralesional steroid injection aided in attaining decannulation. Importantly, the characteristics of stenosis did not significantly affect the outcomes of decannulation.	The BMI was a significant negative predictor of decannulation success. Age was a significant negative predictor of decannulation success. Comorbid pulmonary disease trended toward being a negative predictor. Gender, comorbid pulmonary disease, comorbidity score, COVID-19 as the cause of TCT, and a history of autoimmune disease did not significantly impact decannulation outcomes. Stenosis characteristics, such as tracheomalacia, EDAC, multilevel stenosis, posterior glottic stenosis, and anterior granulation tissue shelf, did not significantly affect decannulation success.
Chauhan et al. 2020 [77] Location: Department of Otolaryngology—Head and Neck Surgery, Chandigarh. Country: India.	To assess elements influencing TCT decannulation in children.	Study design: prospective observational study. Size: 67 patients. Age: mean age of 4.88 ± 3.70 years. Sex: 22 females and 45 males. Diagnosis: pediatric patients requiring TCT decannulation.	January 2014 to April 2015.	Data sources: patient medical records, chest and soft tissue neck X-rays, bronchoscopic assessments, and stroboscopic evaluation. Follow-up: office-based follow-up evaluation one month after decannulation trial. All patients were followed up until the end of the study period.	TCT decannulation success; condition of stoma; swallowing function; breathing status; phonation status; cough reflexes.	The reason for and length of TCT significantly influenced decannulation results, with bronchoscopic evaluations and X-ray results closely related to success. Extended mechanical ventilation was the primary reason for TCT placement, and a gradual, staged decannulation was shown to be safe for pediatric patients. A period of one month was generally sufficient to evaluate the results of the first decannulation attempt, and the length of previous intubation and mechanical ventilation had little impact on the decannulation results.	TCT indication of airway narrowing/stenosis was linked to higher failed decannulation rates. Prolonged mechanical ventilation and acute non-intubatable airway obstruction reduced failure chances. Extended TCT duration increased failure likelihood. X-ray and bronchoscopic assessments revealed abnormal findings correlated with failed decannulation. Age, prior intubation duration, ventilation duration, tube type, cultures, and antibiotics did not significantly affect success.
Krebs et al. 2021 [78] Location: a single tertiary care institution. Country: United States.	To examine the duration of TCT, factors predicting decannulation, and long-term survival rates in patients receiving cardiac surgery.	Study design: retrospective study. Size: 14,600 patients underwent cardiac surgery, with 309 patients requiring TCT. Age: median age of patients requiring TCT—68 years; median age of patients not requiring TCT—65 years; median age of decannulated patients—67 years; median age of non-decannulated patients—70 years. Sex: not specified. Diagnosis: patients undergoing cardiac surgery who required TCT within 60 days of surgery.	1997 to 2016.	Data sources: Institutional Society of Thoracic Surgeons database and electronic medical records database. Follow-up: long-term follow-up was conducted to assess survival and decannulation.	TCT decannulation; overall survival; time to decannulation.	The median duration until TCT decannulation was 59 days, with an 80% decannulation rate after one year in surviving patients. Nonetheless, patients needing a TCT experienced worse long-term survival. Factors like advanced age, chronic lung conditions, and preoperative or postoperative dialysis were associated with reduced decannulation rates. Even with these risk factors, TCT itself did not forecast higher long-term mortality when controlling for other variables.	Older age: negative predictor. Chronic lung disease: negative predictor. Preoperative/postoperative dialysis: negative predictor. Younger age: positive predictor. Lack of chronic pulmonary disease: positive predictor.
Küchler et al. 2019 [79] Location: a hospital. Country: not specified.	To examine the timeline of decannulation and the success rate of decannulation in individuals with sSAH. To determine the factors related to the duration until decannulation and the failure of decannulation.	Study design: retrospective study. Size: 87 patients. Age: median age of 56 years. Sex: 64 females (73.6%); 23 males (26.4%). Diagnosis: sSAH with World Federation of Neurosurgical Societies grade 3–5, requiring TCT due to prolonged mechanical ventilation.	Not specified.	Data sources: hospital medical database. Follow-up: 200 days after TCT. Functional outcome was also assessed at discharge, 3 months, and 6 months.	Time to decannulation; decannulation failure; modified Rankin Scale for functional outcome.	Decannulation was successfully performed in 84% of patients, with a median duration to decannulation of 47 days. Advanced age, a low WFNS grade (IV–V), decompressive craniectomy, and pneumonia were each linked to an increased duration before decannulation. Every patient who faced decannulation failure had poor outcomes. Moreover, pneumonia occurred significantly more frequently in the decannulation failure group.	Negative predictors (longer time to decannulation): older age; poor WFNS grade (IV–V); decompressive craniectomy; and pneumonia. WFNS grade III was associated with early decannulation. Chronic lung disease trended towards negative prediction but was not statistically significant.
Ou et al. 2025 [80] Location: Rehabilitation department of the second affiliated hospital of Kunming Medical University. Country: China.	To create a predictive model (nomogram) that evaluates the probability of decannulation in TCT individuals following neurological trauma. To determine elements that indicate successful decannulation.	Study design: retrospective study. Size: 186 patients. Age: patients aged 18 years and older. Patients were divided into two groups, those younger than 60 and those 60 and older. Sex: cannulated group (n = 63)—50 male (79.37%); 13 female (20.63%); decannulated group (n = 123)—90 male (73.17%); 33 female (26.83%). Diagnosis: neurological injury (stroke or TBI) requiring TCT.	January 2018 to March 2021.	Data sources: medical records. Follow-up: of the decannulated subjects, 91.87% were decannulated within 3 months.	TCT decannulation’ GCS score; swallowing function; Blood indicators (procalcitonin, white blood cell count, hemoglobin, and albumin.	A predictive nomogram was developed to evaluate the probability of successful decannulation. Younger age, female gender, TBI, elevated GCS scores, intact swallowing function, reduced TCT duration, decreased procalcitonin levels, normal white blood cell count, and increased albumin levels were linked to greater decannulation rates. In total, 66.13% of patients underwent successful decannulation, with 91.87% accomplishing it within a three-month period. The nomogram showed significant discriminative capability and practical clinical value.	Age < 60 years. Female sex. TBI. GCS ≥ 8. Normal swallowing function. TCT duration ≤ 3 months. Procalcitonin ≤ 0.05. Normal white blood cell count. Albumin ≥ 35 g/L.
Weyh et al. 2020 [81] Location: University of Florida Jacksonville. Country: United States.	To assess results related to obese patients receiving TCT, concentrating on the timeframe from ventilator liberation to 30 days after decannulation. To assess the rate of successful TCT downsizing and decannulation. To determine the pre-, intra-, and post-operative factors linked to successful TCT downsizing and decannulation. To develop a checklist for downsizing.	Study design: retrospective study. Size: 82 subjects with BMI ≥ 30 (obese group); 173 subjects with BMI < 30 (non-obese group). Age: 15–88 years. Sex: 167 (65%) males; 121 (70%) females. Diagnosis: patients who underwent TCT (open or percutaneous).	April 2016 to December 2018.	Data sources: electronic medical records. Follow-up: 30 days post decannulation and 1 year post TCT.	Successful TCT decannulation; successful TCT downsize; total duration of TCT dependence; Charlson comorbidity index.	Obese individuals had a higher likelihood of staying TCT-dependent for a longer time, experiencing an extended period before decannulation. Although the overall rates of downsize and decannulation were high, they were notably lower in obese individuals than in non-obese patients, with super morbid obesity exhibiting especially poor success rates for downsizing. An elevated BMI correlated with a longer duration until decannulation, and issues like complicated diabetes, chronic kidney disease, chronic obstructive pulmonary disease, and obstructive sleep apnea further heightened the risk of downsizing failure. A structured checklist for downsizing was created to enhance management efficiency.	Increased BMI: negative predictor (increased TCT dependence). Complicated diabetes: negative predictor (increased odds of downsize failure). Chronic kidney disease: negative predictor (increased odds of downsize failure). Chronic obstructive lung disease: negative predictor (increased odds of downsize failure). Obstructive sleep apnea: negative predictor (increased odds of downsize failure and increased time to decannulation). Higher comorbidity score: negative predictor (increased odds of long-term TCT dependence).
Pozzi et al. 2017 [82] Location: a neurological rehabilitation unit. Country: not specified.	To assess the efficacy of a decannulation procedure for young patients with sABI. To determine elements linked to the success and timing of decannulation. To confirm the actual outcomes of their decannulation protocol in their clinical work.	Study design: retrospective study. Size: 123 children. Age: children aged 0–17 years; median age of 8.7 years. Sex: 45 (36.3%) females. Diagnosis: sABI requiring TCT. Etiologies include trauma, hypoxia, brain tumor, stroke, and brain infection.	Data collected from 2002 to 2016 (15 years).	Data sources: not specified. Follow-up: average of 3.8 ± 3.5 years.	Decannulation success (absence of need for recannulation within 1 month); decannulation timing (first rehabilitation stay vs. follow-up); GCS; Level of Cognitive Function; Glasgow Outcome Scale; FIM; DRS; oxymetric monitoring; microbiological cultures; fiberoptic laryngoscopy; polysomnography; optoelectronic plethysmography.	The decannulation protocol was exceptionally successful, with no patients who adhered to it experiencing failures. The timing of decannulation was affected by age, the cause of brain injury, and whether respiratory complications and dysphagia were present. Critical elements influencing the success of decannulation comprised GCS, neurosurgical requirements, and ongoing respiratory problems. Breath-holding spells linked to age and persistent respiratory issues considerably slowed the progress. The protocol emphasized an extensive evaluation of respiratory capability and secretion control to guarantee safe decannulation.	Positive predictors: higher GCS scores; TBI (compared to hypoxic injury); improved rehabilitation outcomes (FIM and DRS); absence of persistent dysphagia; and absence of persistent respiratory complications. Negative predictors: hypoxic brain injury; need for neurosurgery; persistent respiratory complications; persistent dysphagia; and age below 5.5 years (risk of breath-holding spells).
Qin et al. 2025 [83] Location: a tertiary grade A hospital. Country: China.	To determine the independent factors that predict successful decannulation in stroke patients who received TCT. To assess the forecasting capacity of the GCS score, MEBDT, and cough effectiveness for successful decannulation.	Study design: retrospective study. Size: 219 patients. Age: mean age of 54.32 ± 14.96 years. Sex: 155 (70.8%) male and 64 (29.2%) female. Diagnosis: stroke (ischemic or hemorrhagic) confirmed through brain computed tomography or magnetic resonance imaging, requiring TCT.	January 2020 to December 2023.	Data sources: medical records, rehabilitation assessments, and laboratory test results. Follow-up: 7 days after the TCT tube is removed. Decannulation status was evaluated within 3 months post TCT.	Successful decannulation (no re-intubation within 7 days); GCS scorel MEBDT; coughing ability; nutritional risk; presence of multidrug-resistant bacteria in sputum; white blood cell count; fiberoptic bronchoscopy.	The GCS score, MEBDT outcomes, and coughing ability independently affected TCT decannulation. A positive MEBDT outcome was a major risk factor for failing decannulation, whereas elevated GCS scores and robust cough capability were favorable factors for achieving success. When put together, these three measures showed great predictive ability for decannulation results. Three months after TCT, the decannulation rate was 31.5% overall.	Positive predictors: high GCS scores; strong cough ability; and negative MEBDT. Negative predictors: positive MEBDT.
Cheng et al. 2024 [84] Location: multicenter, international clinical study. Country: multiple countries.	To determine the predictive factors for successful PES treatment in dysphagic stroke patients needing mechanical ventilation and TCT. To examine the connection between predictor variables and the likelihood of decannulation.	Study design: retrospective study. Size: 98 participants; 60 participants received PES during TCT. Age: mean age of 66.6 (13.0) years. Sex: 72 (73.5%) male and 26 (26.5%) female. Diagnosis: oropharyngeal dysphagia in stroke patients requiring mechanical ventilation and TCT.	Data collected from the PHADER study, which took place between March 2015 and September 2018.	Data sources: data collected from the PHADER study. Follow-up: assessments were performed at baseline, day 5, day 9, and 3 months (day 92) post treatment.	DSRS and NIHSS.	The prompt start of PES therapy and being younger were crucial indicators of success. Individuals with supratentorial strokes and those who were receiving nasogastric or nasojejunal feeding at the start had an increased chance of favorable outcomes. Moreover, a reduced duration from stroke onset to PES notably enhanced the likelihood of successful decannulation.	Positive predictors: shorter time from stroke onset to PES.
Obayashi et al. 2023 [85] Location: Shizuoka Children’s Hospital. Country: Japan.	To review in hindsight the results of a gradual TCT decannulation program in children. To assess the safety and efficacy of the decannulation procedure. To outline the causes of unsuccessful decannulation.	Study design: retrospective study. Size: 77 patients (86 decannulation trials). Age: mean age at first decannulation protocol of 6.5 ± 3.6 years. Sex: 41 (53.2%) male and 36 (46.8%) female. Diagnosis: various conditions requiring TCT, including bilateral vocal cord paralysis, bronchomalacia, subglottic stenosis, and others.	January 2011 to November 2022.	Data sources: medical records. Follow-up: outpatient follow-up for over 6 months	Decannulation success rate; failure rate in each phase of the decannulation program; clinical summaries of failed decannulation cases.	The total success rate for decannulation was 90.9%, although failures happened at various stages, highlighting the necessity for a gradual strategy. Major causes of failure involved respiratory distress, desaturation, and issues associated with closing the tracheocutaneous fistula. To tackle these issues, a two-phase surgical method for the closure of tracheocutaneous fistulas was introduced. This underscores the significance of customized approaches to enhance patient results.	Factors related to success: meeting eligibility criteria (no ventilation support, no aspiration, patent airway, and successful capping trials). Factors related to failure: respiratory distress during capping trials; desaturation; stridor; dyspnea; complications from TCF closure (bradycardia and asphyxia); and underlying conditions such as glossoptosis, tracheomalacia, and subglottic stenosis.
Muhle et al. 2021 [86] Location: Neurological ICU at Münster University Hospital. Country: Germany.	To evaluate the safety and effectiveness of the “Standardized endoscopic Swallowing Evaluation for TCT decannulation in critically ill neurological patients” (SESETD). To determine the factors that predict (early) decannulation and failure of decannulation.	Study design: prospective observational study. Size: 377 patients. Age: mean age of 62.6 ± 15.7 years. Sex: 158 females and 219 males. Diagnosis: various neurological conditions requiring TCT, including ischemic stroke, hemorrhagic stroke, Guillain-Barré syndrome, meningitis, and myopathy.	January 2013 to December 2017.	Data sources: patient medical records and clinical assessments. Follow-up: during the patient’s stay in the ICU.	Decannulation success and failure; SESETD score; mRS; RASS; FOIS; FEES.	The SESETD demonstrated itself as a reliable and efficient instrument for directing decannulation choices, featuring a minimal failure rate of only 3.5%. A prolonged period of mechanical ventilation was a significant factor in predicting decannulation failure, whereas a younger age correlated with sooner decannulation. Furthermore, the initial SESETD score was a strong predictor of successful decannulation during the ICU admission, emphasizing its clinical relevance.	Positive predictors: higher initial SESETD score and younger age for early decannulation. Negative predictors: longer duration of mechanical ventilation (predictive of decannulation failure).
Sillers et al. 2022 [87] Location: Children’s Hospital of Philadelphia, Philadelphia Country: United States.	To characterize the rates of mortality and decannulation in early childhood for infants who underwent TCT in their first year. To evaluate results following infant TCT depending on the specific reason for TCT insertion.	Study design: retrospective study. Size: 378 infants. Age: median postmenstrual age at birth of 35.5 weeks; median chronological age at TCT of 129 days. Sex: 225 males (59.5%); 153 females (40.5%). Diagnosis: primary indications for TCT included pulmonary diagnoses (e.g., chronic lung disease), anatomic diagnoses, cardiac diagnoses, and neurologic/musculoskeletal diagnoses.	1 January 2001 to 1 May 2013 (TCT placement).	Data sources: clinical records (inpatient medical records, outpatient otolaryngology clinic notes), surgical database (OR manager), and billing codes. Follow-up: until successful decannulation, documentation of death, or completion of the study period (17 May 2018). Post-discharge analyses included infants with at least three years of clinical data after TCT placement.	In-hospital mortality; post-discharge mortality; decannulation rates; respiratory support at discharge.	Results differed considerably based on the main motive for TCT. Infants with neurological or musculoskeletal conditions experienced the lowest rates of decannulation, whereas very premature infants exhibited reduced overall mortality and enhanced likelihood of decannulation. Male gender, greater postmenstrual age at birth, and increased age at surgery were associated with reduced survival chances. Moreover, certain neurologic or musculoskeletal conditions and specific racial or ethnic groups were individually linked to decreased survival and decannulation rates.	Negative predictors: neurologic or musculoskeletal diagnoses; older postmenstrual age at birth; and a racial or ethnic background other than black, non-Hispanic white, or Hispanic. Positive predictors: very premature birth.
Aljedaani et al. 2020 [88] Location: a tertiary hospital in the south of Jeddah, Saudi Arabia. Country: Saudi Arabia.	To evaluate the decannulation procedure and its success rate in individuals who have had TCT.	Study design: prospective observational study. Size: 102 patients were initially included, with 87 patients analyzed after exclusions. Age: mean adult age ranged from 57.1 to 65.0 years, depending on the TCT method and sex; mean pediatric age of 9.54 years. Sex: 42 adult males (48%), 37 adult females (43%), and 8 pediatric patients (9%). Diagnosis: patients needing TCT due to diverse underlying conditions that require airway management.	October 2016 to October 2018.	Data sources: patient medical records and clinical assessments. Follow-up: 48 h post decannulation to monitor for decannulation failure.	Successful and unsuccessful decannulation; evaluation of decannulation standards (alertness, breathing capability, swallowing skill, coughing efficiency, and airway openness); monitoring symptoms and indicators of failure such as dyspnea, stridor, tachypnea, tachycardia, and decrease in oxygen saturation.	Decannulation was achieved successfully in 97.7% of instances, with merely two patients (2.3%) encountering failure. These results endorse the efficacy of a protocol-driven method in facilitating safe and effective decannulation. The research emphasizes the significance of organized clinical decision-making to improve patient results.	Positive predictors for TCT success include a conscious and alert state, resolution of the primary condition, independence from ventilator support, effective cough, oxygen saturation ≥ 97%, safe swallowing, confirmed patent airway, and tolerance of tube capping for ≥72 h. Negative predictors comprise obesity, comorbidities, pneumonia, nocturnal desaturation, altered mental status, weak cough reflex, suctioning needs, impaired swallowing, and upper airway obstruction.
Quinlan et al. 2019 [89] Location: Children’s Hospital of Philadelphia. Country: United States.	To assess the function of PSG in individuals with BPD and TCT who are going through decannulation. To evaluate the decannulation success rate between individuals who underwent pre-decannulation PSG and those who did not receive it.	Study design: retrospective study. Size: 125 patients deemed clinically eligible for TCT decannulation. Age: median age at decannulation ranged from 4.41 to 5.07 years; median age at PSG ranged from 4.04 to 6.04 years. Sex: 57% male. Diagnosis: BPD and/or chronic lung disease of prematurity.	1 January 2007 to 1 June 2017.	Data sources: electronic patient records; Sleep Lab database; and billing system. Follow-up: successful decannulation was defined as being decannulated for 6 months without replacement of the TCT tube. TCT decannulation failure was defined as reinsertion of the TCT tube within 6 months of decannulation trial.	Decannulation success and failure; PSG variables (obstructive apnea–hypopnea index and central apnea–hypopnea index).	Patients with BPD and a TCT experienced a high success rate in decannulation, irrespective of whether they had a pre-decannulation PSG. There was no notable difference in success rates between the groups, but participants who underwent PSG without a decannulation trial exhibited higher OAHI, maximum ETCO2, and gestational age. PSG was instrumental in influencing decannulation choices by detecting obstructive sleep apnea. These results emphasize the importance of PSG in improving patient outcomes.	Positive factors: passing the decannulation trial. Negative factors: higher OAHI in PSG; higher ETCO2 max in PSG; uncontrolled seizure disorder; requirement for repeat laryngotracheal reconstruction; recurrence of subglottic stenosis; and genetic comorbidities.
Thomas et al. 2017 [90] Location: post-acute ICU and rehabilitation units. Country: Germany.	To outline the timing and related risk factors of removing the TCT tube in chronically critically ill patients with ICU-acquired weakness during their rehabilitation process.	Study design: prospective cohort study. Size: 122 patients. Age: 18 years old. Sex: not specified. Diagnosis: ICU-acquired weakness, muscle weakness pathology (e.g., critical illness myopathy and polyneuropathy), and chronic critical illness (defined as >21 days ICU treatment including mechanical ventilation and at least 14 more days of ICU treatment with or without mechanical ventilation).	January 2013 to March 2015 (recruitment period).	Data sources: patient medical records, clinical assessments, and standardized measures administered by trained therapists. Follow-up: one year or until successful decannulation, whichever was sooner.	Successful decannulation; time to decannulation; activities of daily living (Barthel Index). clinical severity (Early Rehabilitation Barthel Index and APACHEII Score); number of indwelling catheters and cannulas; muscle strength (MRC and grip strength); functional status score for the intensive care unit; Physical Function ICU Test; pain (numeric pain rating scale); balance (functional reach); cognitive measures (Montreal Cognitive Assessment).	A successful decannulation was accomplished after a median of 40.5 days since the study began and 89 days since the start of the primary illness. The quantity of medical tubes and the length of ventilator weaning were important indicators of successful decannulation. A lower number of indwelling medical devices and reduced mechanical ventilation durations were linked to increased decannulation rates. Importantly, no negative events were observed after decannulation, underscoring the safety of the procedure.	Negative predictors: higher number of indwelling medical tubes (catheters) and longer duration of mechanical ventilation/weaning. Positive predictors: lower number of indwelling medical tubes and shorter duration of mechanical ventilation/weaning.
Beaton et al. 2016 [91] Location: Royal Hospital for Sick Children in Glasgow. Country: Scotland.	To evaluate the results of a TCT decannulation protocol implemented in a pediatric ward. To assess the effectiveness and safety of the decannulation procedure.	Study design: retrospective study. Size: 45 patients. Age: age at original TCT of 1 day to 16 years and 6 months (median 3 months); age at decannulation of 6 months to 16 years and 8 months (median 2 years and 6 months). Sex: 25 male (56%) and 20 female (44%). Diagnosis: various indications for TCT, including tracheobronchomalacia, bilateral vocal cord palsy, subglottic stenosis, and others.	January 2012 to May 2015.	Data sources: patient medical records. Follow-up: follow-up was implied by the data collection of reinsertion of TCT tubes after decannulation.	Success or failure of ward decannulation; timing of decannulation; patient characteristics; endoscopic airway assessment results; overnight sleep study results.	Decannulation was accomplished on the initial attempt in 58% of instances and 73% in total. The primary failure point occurred on Day 2 of the protocol, during the capping of the TCT tube, with suprastomal malacia, adenotonsillar hypertrophy, and chest infections as the main contributors. Decannulation age and weight did not significantly influence success rates. Crucially, none of the negative events necessitated emergency airway intervention, underscoring the safety of the procedure.	Positive Factors: resolution of underlying airway pathology; favorable endoscopic airway assessment; and satisfactory overnight sleep study results. Negative factors: suprastomal malacia; adenotonsillar hypertrophy; chest infection; and bilateral vocal cord palsy, due to difficulty in assessing improvement.
Lui et al. 2024 [92] Location: Prince of Wales Hospital, Hong Kong. Country: China.	To determine the predictive factors linked to challenging TCT decannulation in neurosurgery patients. To assess how the challenges of decannulation affect neurological and hospital results.	Study design: retrospective study. Size: 131 TCT neurosurgical patients. Age: mean age at TCT of 59 ± 11 years. Sex: 78 male (60%). Diagnosis: neurosurgical conditions requiring TCT, including stroke-related conditions and traumatic brain injury.	1 September 2016 to 31 August 2019.	Data sources: retrospective review of patient medical records. Follow-up: at least one year.	Easy decannulation (within 3 months) vs. difficult decannulation (inability to decannulate within 3 months); GCS on admission and discharge; vocal cord palsy; pneumonia within 1 month post TCT; Glasgow Outcome Score at 6 months and 1 year; length of inpatient stay. mobility upon discharge; destination after discharge.	A GCS of 8 or less at admission, vocal cord paralysis noted at 3 months, and pneumonia developing within 1 month post TCT were closely associated with difficult decannulation. Difficulties with decannulation were associated with longer hospital stays, lower GCS at discharge, poorer neurological outcomes, and a higher likelihood of being discharged to a nursing home. The GCS motor score had a stronger correlation with the difficulties of decannulation compared to the verbal score.	Negative predictors: GCS ≤ 8 on admission; vocal cord palsy at 3 months post TCT; and pneumonia within 1 month post TCT. Positive predictors: higher GCS scores on admission; absence of vocal cord palsy; and absence of pneumonia post TCT.

Legend: tracheostomy (TCT); intensive care units (ICUs); Acute Physiology and Chronic Health Evaluation II (APACHE II); severe acquired brain injury (sABI); Glasgow Coma Scale (GCS); body mass index (BMI); oxygen saturation (SpO2); mean inspiratory pressure (MIP); mean expiratory pressure (MEP); fiberoptic endoscopic evaluation of swallowing (FEES); Murray Secretion Scale (MSS); National Institutes of Health Stroke Scale (NIHSS); peak cough flow (PCF); peak expiratory Flow (PEF); blue dye test (BDT); Videofluoroscopic Swallowing Study (VFSS); early rehabilitation care (ERC); Coma Recovery Scale—Revised (CRS-R); hyper-acute rehabilitation unit (HARU); Functional Independence Measure (FIM); Functional Assessment Measure (FAM); paroxysmal sympathetic hyperactivity (PSH); length of stay (LOS); Functional Oral Intake Scale (FOIS); acquired brain injury (ABI); Early Functional Abilities (EFA); traumatic brain injury (TBI); Palliative Care Center (PCC); cervical spinal cord injury (CSCI); American Spinal Injury Association impairment scale (AIS); neurological level of injury (NLI); Charlson comorbidity index (CCI); functional dysphagia scale (FDS); penetration aspiration scale (PAS); Korean version of Mini-Mental State Examination (K-MMSE); Korean version of the modified Barthel Index (K-MBI); activities of daily living (ADL); unassisted peak cough flow (UPCF); assisted peak cough flow (APCF); assisted peak cough flow with external control device (APCF-ECD); cough flow strength value (CFSV); ventilatory pump failure (VPF); Coma Recovery Scale–Revised (CRS-R); Disability Rating Scale (DRS); Level of Cognitive Function (LCF); Minimally Conscious State (MCS); emergence from Minimally Conscious State (E-MCS); unresponsive wakefulness syndrome (UWS); modified Rankin Scale (mRS); Airway Care Score (ACS); computer tomography (CT); magnetic resonance imaging (MRI); disorders of consciousness (DoC); persistent vegetative state (PVS); decision curve analysis (DCA); Receiver Operating Characteristic (ROC); fiberoptic laryngoscopy (FOL); Saturation of Peripheral Oxygen (SpO2); Injury Severity Score (ISS); Cerebral Performance Category (CPC); capped tracheostomy tube polysomnography (cPSG); bronchopulmonary dysplasia (BPD); apnea hypopnea index (AHI); end-tidal carbon dioxide (ETCO2); prolonged disorders of consciousness (pDoC); electroencephalography (EEG); pharyngeal electrical stimulation (PES); Trial of ORG 10172 in Acute Stroke Treatment (TOAST); posterior glottic stenosis (PGS); Guillain–Barré syndrome (GBS); Erasmus GBS Respiratory Insufficiency Score (EGRIS); peristomal subglottic stenosis (SGS); Excessive Dynamic Airway Collapse (EDAC); carbon dioxide (CO_2_); severe subarachnoid hemorrhage (sSAH); World Federation of Neurosurgical Societies (WFNS); modified Evan’s blue dye test (MEBDT); Dysphagia Severity Rating Scale (DSRS); Richmond Agitation–Sedation Scale (RASS); polysomnography (PSG); obstructive apnea–hypopnea index (OAHI); end-tidal carbon dioxide (ETCO2).

## 4. Discussion

### 4.1. Qualitative Analysis

The landscape of TCT decannulation, as illustrated by the provided studies, shows a complicated interaction of patient demographics, clinical factors, and methodological strategies. A thorough assessment requires a detailed comprehension of these elements to guide evidence-based medical practice. Initially, the diversity of patient groups in these studies is a notable characteristic. Research covers various clinical contexts, including sABI [44,45,46], stroke [55,61], spinal cord injuries [54], and general intensive care environments [43]. This variability, although indicative of the wide applicability of TCT, makes it difficult to synthesize findings and create universal decannulation protocols. The difficulty is in identifying consistently predictive elements among these varied groups. Additionally, the lack of uniform decannulation protocols is a common issue. Research utilizes different approaches, such as limiting trials, regulating suctioning frequency [43], and comprehensive evaluations [47]. This variation highlights the necessity for guidelines based on evidence. Although certain studies support the use of physiological indicators such as PCF [58] and MEP [44], others focus on functional evaluations of swallowing and coughing [56]. The best combination of these strategies continues to be a matter of active discussion. The recognition of predictors for effective decannulation is a key emphasis. Younger individuals, elevated consciousness levels (reflected in GCS scores), and a lack of respiratory issues regularly stand out as important predictors [60,63]. Nonetheless, the significance of these elements differs, indicating the varied patient groups and research methods. Significantly, the use of machine learning models in various studies highlights the possibility of utilizing advanced analytics to improve predictive precision [59]. However, these models need thorough external validation to confirm their clinical usefulness. Additionally, certain studies indicate that elevated BMI poses a risk for laryngeal lesions that hinder decannulation [74]. Complications like aspiration pneumonia and ongoing dysphagia pose major obstacles to effective decannulation. The effect of these issues on hospital stay duration and functional results emphasizes the significance of careful patient selection and collaborative care. The documented decannulation rates differ significantly, highlighting the necessity for personalized strategies that cater to the unique requirements of each patient. From a methodological standpoint, the prevalence of retrospective studies brings about intrinsic limitations, such as possible selection bias and difficulties in determining causality. Although less frequent, prospective studies provide stronger evidence [47,58]. The differences in sample sizes among studies also deserve attention, as they impact statistical power and the applicability of results. Key factors to consider are the effects of obesity, which is known to elevate the likelihood of laryngeal injuries that hinder decannulation [74], and the significance of prompt rehabilitation, which has been indicated to reduce decannulation duration [51]. The application of external control devices to enhance cough effectiveness in individuals with weak coughs is a promising research area as well [57]. In conclusion, although the existing research offers important perspectives on TCT decannulation, additional high-caliber, prospective studies are crucial for creating standardized protocols, enhancing predictive models, and improving patient outcomes via multidisciplinary care.

### 4.2. Decannulation Strategies: Bridging Research to Clinical Practice

The implementation of TCT decannulation protocols from research into clinical practice requires a careful equilibrium between uniform procedures and tailored patient care. Although protocols that include physiological and functional evaluations, like cough strength [44], secretion management [45], and swallowing assessments [73], show potential, their effectiveness differs greatly among patient groups, such as those with sABI [60], stroke [84], and pediatric conditions [64]. This variability highlights the significance of advancing to personalized, data-informed methods. Based on the recognition that structured suctioning and ongoing high-flow oxygen strategies enhance decannulation results and recognizing the proven adverse effects of older age and comorbid conditions on decannulation success, the field is increasingly aimed at improving risk stratification. Predictive models, such as nomograms and machine learning tools, are being created to assess the likelihood and timing of decannulation [93]. These models, represented by advanced DecaPreT models for sABI [67] and those created for persistent vegetative state [65], seek to enhance patient selection. Nonetheless, the fundamental constraints of retrospective data emphasize the essential requirement for prospective validation and external verification of these predictive instruments. Additionally, the beneficial impact of early rehabilitation, especially ICU-related verticalization, on decreasing decannulation duration and enhancing neurological condition underscores the increasing focus on early mobilization for critically ill patients [94,95]. Alongside this, clinical signs like a strong cough, efficient secretion management, and the speaking valve tolerance test continue to be vital elements of clinical evaluation, in accordance with recognized best practices. The efficacy of methods such as pharyngeal electrical stimulation (PES) [73,84] in improving swallowing function, combined with the acknowledged influence of variables like obesity [74,76,81], age [78,79,80], and comorbidities [68,69], highlights the necessity for personalized, patient-centered approaches. Although protocol-driven methods show high success rates [47,77,82,85,88], the ongoing issue of decannulation failure, frequently associated with dysphagia and respiratory issues, requires continual refinement of protocols. From a clinical viewpoint, effectively implementing TCT decannulation protocols into standard practice requires a collaborative strategy that unites respiratory therapists, speech and language therapists, and critical care experts [96]. Utilizing standardized assessment instruments like the modified Evan’s blue dye test for aspiration risk and high-resolution manometry for swallowing abilities can improve the precision of decision-making [97]. Additionally, practical application necessitates flexibility to consider organizational resources and individual patient factors. For example, although PES appears promising, the availability of this technology might be restricted in some environments, requiring other compensatory approaches like enhanced dysphagia therapy and respiratory muscle training [98]. In the end, achieving better decannulation results necessitates a collaborative strategy that combines advanced predictive models with comprehensive clinical assessments, steering clear of excessive dependence on algorithms. This method also encourages essential conversations about the ethical consequences of machine learning in clinical decision-making and highlights the significance of collaborative efforts in creating tailored decannulation plans.

### 4.3. Future Research Directions in Tracheostomy Decannulation

Following this foundation, future studies should focus on large, prospective, multicenter trials to generate firm, evidence-based decannulation regimens applicable in heterogeneous groups of patients. Such studies should investigate the long-term effects of different decannulation regimens, including the incorporation of newer modalities like advanced imaging and biofeedback systems, on durable function and quality of life. Further, more intense study of the pathophysiologic factors leading to decannulation failure, especially in the context of vulnerable groups like the elderly and complex comorbidities, must be undertaken. Elucidation of the role of predictors like biomarkers and genetic predisposition may perhaps hold the secrets to personalized treatments. With the encouraging but nascent experience with machine learning models, external validation and model refinement with the inclusion of real-time data and dynamic patient variables become imperative. Addressing issues of algorithmic bias and data privacy must actively take place to guarantee equality of access and responsible deployment. Standardized, patient-reported scales for the assessment of the patient experience of decannulation and its effect on daily functioning must be developed. How do we better incorporate patient-centered principles of care into decannulation regimens to promote patient empowerment and shared decision-making? And what interventions promote greater interdisciplinary integration to ease the transition from acute to post-acute care environments, with continuity of care and avoidance of complications? Finally, an examination of the economic burden of different decannulation regimes, such as cost-effective analyses of newer interventions, may provide data for healthcare policy-making and resource planning.

### 4.4. Strengths and Limitations

This scoping review highlights numerous significant strengths in its methodology for integrating the literature on predictive factors for effective TCT decannulation. The stringent methodology, defined by an extensive search approach across six key databases, guarantees a wide collection of pertinent studies. Utilizing the PICO framework and outlining distinct inclusion and exclusion criteria improves the clarity and repeatability of the review. The two separate review processes, along with the evaluation of inter-rater reliability using the kappa statistic, reduce possible biases and enhance the validity of the results. Moreover, narrative synthesis, utilized because of the diversity of the included studies, promotes a comprehensive understanding of the intricate interaction of physiological, clinical, and demographic elements affecting decannulation results. The involvement of both adult and pediatric groups, combined with an in-depth examination of demographic and etiological traits, offers a thorough insight into the area. The comprehensive overview of the indications for TCT and the variability in decannulation processes, as influenced by factors such as patient age, underlying diagnoses, and procedural techniques (surgical vs. percutaneous), enhances the clinical relevance of the review. The emphasis on the importance of a systematic approach to decannulation, moving beyond a simplistic view of tube removal, underscores the complexity of patient management in this context. Furthermore, the review effectively identifies and articulates the gaps in the existing literature, particularly the lack of a robust, integrated predictive model.

Despite its strengths, this scoping review exhibits certain limitations that warrant consideration. Firstly, the exclusion of non-English language studies may introduce language bias, potentially overlooking valuable research from diverse international contexts. Secondly, the reliance on narrative synthesis, while appropriate for the heterogeneity of the data, may limit the ability to conduct quantitative analyses, thereby precluding the application of meta-analytic techniques to derive pooled effect sizes. The inherent limitations of retrospective studies, which comprise a significant portion of the included literature, such as potential selection bias and incomplete data, should also be acknowledged. The variability in study designs, observation periods, and data collection tools across the included studies introduces heterogeneity that can complicate the synthesis of findings.

## 5. Conclusions

In conclusion, this scoping review has revealed the complex web of factors affecting TCT decannulation. It highlights that successful decannulation is not an isolated occurrence but instead a result of various physiological, clinical, and demographic factors. Significantly, strong respiratory function, demonstrated by powerful cough reflexes and efficient secretion control, stands out as a fundamental aspect. Neurological stability, especially demonstrated by elevated GCS scores and the lack of severe encephalopathy, is also vital. Age and the presence of comorbidities consistently influence decannulation outcomes, with a younger age and fewer comorbidities enhancing the likelihood of success. The transition to objective measures, including PCF and standardized swallowing evaluations, indicates progression toward more accurate clinical decision-making. Additionally, the significance of thorough rehabilitation and the removal of airway blockages cannot be exaggerated. This review underscores the importance of personalized, multidisciplinary strategies for decannulation, stressing the requirement for prospective research to confirm these results and improve clinical guidelines, thereby enhancing patient recovery and results. Given these results, clinicians are recommended to incorporate structured interdisciplinary decannulation protocols that include objective assessments of respiratory and neurologic status, peak cough flow, swallowing assessments, and GCS scoring as routine practice. These interventions may help standardize decision-making, decrease outcome variability, and promote safer and more patient-driven decannulation processes. Adding to that, more cooperation between pulmonary physicians, speech-language pathologists, and critical care teams is needed to translate that knowledge into daily practice.

## Figures and Tables

**Table 1:** Structured phases of the decannulation process.

Phase	Description	Key Clinical Considerations
Initial Patient Evaluation [26]	A comprehensive assessment of the patient’s respiratory function, upper airway patency, cough effectiveness, and secretion management [26].	Includes bedside spirometry, arterial blood gas analysis, and multidisciplinary input from respiratory therapists, speech therapists, and pulmonologists [27].
Cuff Deflation Trial [28]	Progressive deflation of the tracheostomy tube cuff to evaluate upper airway competence and detect signs of aspiration or respiratory distress [28].	Close monitoring for stridor, increased work of breathing, and changes in oxygen saturation. Aspiration risk may be assessed using a blue dye test [28].
Tracheostomy Tube Downsizing [29]	Gradual reduction in tube size to encourage upper airway use while maintaining respiratory stability [29].	A stepwise approach with progressively smaller tubes. Respiratory effort and secretion clearance should be carefully monitored [29].
Capping Trials [30]	Temporary occlusion of the tracheostomy tube with a cap or a speaking valve to assess the ability to breathe through the upper airway [30].	Initiated with short intervals and progressively extended. Monitoring includes end-tidal CO_2_ levels and patient comfort [30].
Respiratory Tolerance Assessment [31]	Observation of the patient’s ability to sustain spontaneous breathing without airway obstruction or secretion retention [31].	Key indicators include stable oxygen saturation (>92%) and an effective cough reflex [31].
Swallowing and Aspiration Screening [32]	Evaluation of swallowing function to ensure safe management of oral secretions without aspiration risk [32].	May involve modified barium swallow studies or fiberoptic endoscopic evaluation of swallowing [32].
Final Decannulation Readiness Check [33]	A final assessment to confirm sufficient respiratory function and airway stability before tube removal [33].	Includes pulmonary function tests, arterial blood gas analysis, and confirmation of an adequate breathing pattern [33].
Decannulation Procedure [34]	Removal of the tracheostomy tube, with immediate coverage of the stoma using a sterile dressing and post-removal monitoring [34].	Emergency airway equipment is kept available. The patient is observed in a monitored setting for at least 24 h [34].
Post-Decannulation Monitoring [35]	Continuous assessment for respiratory distress, oxygen desaturation, or airway obstruction following tube removal [35].	Supplemental oxygen may be provided if needed. Monitoring for upper airway collapse and secretion clearance is essential [35].
Stoma Management and Follow-Up [36]	Natural closure of the stoma or surgical intervention if required. Patient education on post-decannulation care and scheduled follow-up assessments [36].	Persistent stomas may require surgical closure. Follow-up evaluations to detect potential complications such as tracheal stenosis or fistula formation [36].

**Table 2:** Detailed summary of the scoping review methodology.

Section Methodology	Key Clinical Considerations
Inclusion Criteria	- Studies including adult and pediatric participants undergoing TCT. - Studies examining physiological, clinical, and demographic factors associated with decannulation outcomes. - Studies specifically addressing predictors of successful decannulation. - Studies reporting outcomes related to time to decannulation, decannulation success rates, and associated complications. - Studies clearly describing the assessed predictors, including methodology, participant demographics, and relevant clinical parameters. - Full-text articles published in English.
Exclusion Criteria	- Studies that do not specifically focus on predictors of successful decannulation in adult and pediatric TCT populations. - Studies investigating non-decannulation-related factors (e.g., general airway management or alternative ventilation techniques). - Studies that do not address relevant decannulation outcomes (e.g., time to decannulation, success rates, and associated complications). - Studies that fail to clearly define TCT participant groups or include mixed airway management populations without isolating TCT patients. - Study protocols, conference abstracts, editorials, and reviews (systematic, narrative, or integrative). - Research published in languages other than English or those without full-text access.
PICO Evaluation	Population: adult and pediatric participants undergoing TCT. Intervention: analysis of various physiological, clinical, and demographic factors potentially predictive of successful decannulation. Comparison: no direct comparison group. Outcome: identification and categorization of key predictors associated with successful decannulation.
Search Period	Search Time Range: from 2014 to 2025. Time Search Conduction: from 22 February 2025 to 3 March 2025.
Study Selection	Two reviewers (A.C. and S.F.) independently screened articles at title, abstract, and full-text levels. Disagreements were resolved through discussion or by a third reviewer (R.S.C.). A PRISMA flowchart used to visualize the selection process [41].
Tool Used	Kappa statistic applied to measure inter-rater agreement (threshold for substantial agreement: kappa > 0.61) [42].
Data Extraction	- Two independent reviewers (A.C. and S.F.) conducted searches to ensure transparency and accuracy. - The PRISMA flowchart was used to depict the study selection process. - Data extraction was performed independently by two reviewers (A.C. and S.F.), with discrepancies resolved by a third reviewer (R.S.C.). - Microsoft Excel facilitated data extraction and organization, ensuring consistency and minimizing error.
Synthesis Approach	Narrative methods were used due to the variety of study designs and interventions. - Key themes, commonalities, and differences were identified across studies. - Studies were grouped by intervention type, relevant predictors of successful decannulation, and reported outcomes. - A multidisciplinary team ensured balanced data interpretation, with consensus meetings to minimize biases and ensure consistency in outcomes categorization.

Legend: tracheostomy (TCT); Preferred Reporting Items for Systematic Reviews and Meta-Analyses (PRISMA).

## Data Availability

Not applicable.
